# Acetylcholine released by endothelial cells facilitates flow‐mediated dilatation

**DOI:** 10.1113/JP272927

**Published:** 2016-12-14

**Authors:** Calum Wilson, Matthew D. Lee, John G. McCarron

**Affiliations:** ^1^ Strathclyde Institute of Pharmacy and Biomedical SciencesUniversity of StrathclydeSIPBS BuildingGlasgowUK

## Abstract

**Key points:**

- The endothelium plays a pivotal role in the vascular response to chemical and mechanical stimuli.
- The endothelium is exquisitely sensitive to ACh, although the physiological significance of ACh‐induced activation of the endothelium is unknown.
- In the present study, we investigated the mechanisms of flow‐mediated endothelial calcium signalling.
- Our data establish that flow‐mediated endothelial calcium responses arise from the autocrine action of non‐neuronal ACh released by the endothelium.

**Abstract:**

Circulating blood generates frictional forces (shear stress) on the walls of blood vessels. These frictional forces critically regulate vascular function. The endothelium senses these frictional forces and, in response, releases various vasodilators that relax smooth muscle cells in a process termed flow‐mediated dilatation. Although some elements of the signalling mechanisms have been identified, precisely how flow is sensed and transduced to cause the release of relaxing factors is poorly understood. By imaging signalling in large areas of the endothelium of intact arteries, we show that the endothelium responds to flow by releasing ACh. Once liberated, ACh acts to trigger calcium release from the internal store in endothelial cells, nitric oxide production and artery relaxation. Flow‐activated release of ACh from the endothelium is non‐vesicular and occurs via organic cation transporters. ACh is generated following mitochondrial production of acetylCoA. Thus, we show ACh is an autocrine signalling molecule released from endothelial cells, and identify a new role for the classical neurotransmitter in endothelial mechanotransduction.

## Key points

- The endothelium plays a pivotal role in the vascular response to chemical and mechanical stimuli.
- The endothelium is exquisitely sensitive to ACh, although the physiological significance of ACh‐induced activation of the endothelium is unknown.
- In the present study, we investigated the mechanisms of flow‐mediated endothelial calcium signalling.
- Our data establish that flow‐mediated endothelial calcium responses arise from the autocrine action of non‐neuronal ACh released by the endothelium.

**Abbreviations**
acetyl‐CoA: acetyl‐coenzyme A
AChE: acetylcholinesterase
2‐APB: 2‐aminoethoxydiphenyl borate
bromoACh: bromoacetylcholine
Cal‐520/AM: Cal‐520 acetoxymethyl ester
CCCP: carbonyl cyanide 3‐chlorophenylhydrazone
CFTR: cystic fibrosis transmembrane regulator
CFTR_inh_172: cystic fibrosis transmembrane regulator inhibitor 172
ChAT: choline acetyltransferase
CPA: cyclopiazonic acid
DAF‐FM: 4‐amino‐5‐methylamino‐2′,7′‐difluorofluorescein diacetate
IP_3_: inositol trisphosphate
IP_3_R: inositol trisphosphate receptor
M3AchR: M3 ACh receptor
OCT: organic cation transporter
PLC: phospholipase C
PSS: physiological saline solution
ROI: region of interest
RuR: ruthenium red
Ry: ryanodine
RyR: ryanodine receptor
TMRE: tetramethylrhodamine ethyl ester
TRP: transient receptor potential
TRPC: transient receptor potential canonical
TRPV: transient receptor potential vanilloid
VAChT: vesicular ACh transporter
ΔΨ_m_: mitochondrial membrane potential

## Introduction

The endothelium is a complex sensory system that acts as an innate mechanotransducer and enables the vascular system to regulate cardiovascular function. The endothelium detects frictional shear stresses generated by the flow of blood (Davies, 1995) and transmits signals to the vascular wall to control flow‐dependent phenomena such as angiogenesis (Kaunas *et al*. 2011), vascular remodelling (Lucitti *et al*. 2007) and the occurrence of disease such as atherosclerosis (Gibson *et al*. 1993). In animals (Cooke *et al*. 1991) and humans (Joannides *et al*. 1995), an increase in blood flow also causes an increase in arterial diameter. This phenomenon, termed flow‐mediated dilatation, arises from endothelium‐dependent relaxation of underlying smooth muscle. However, the mechanisms responsible for the detection of mechanical stimuli by the endothelium, as well as the initiation of flow‐mediated dilatation, are not well understood.

In addition to mechanical stimuli, the endothelium also responds to biochemical signalling molecules to affect a diverse range of vascular functions, such as vascular tone (Furchgott & Zawadzki, 1980) and blood clotting (Stern *et al*. 1991). The significance of the endothelium in the control of vascular tone was first demonstrated by the observation that ACh induced smooth muscle relaxation only in arteries with an intact endothelial layer (Furchgott & Zawadzki, 1980). Subsequently, the endothelium has been shown to regulate vasoactivity, either partially or entirely, by releasing various endothelium‐dependent relaxation factors (e.g. nitric oxide, prostaglandins and endothelium‐derived hyperpolarization factor) (Furchgott & Zawadzki, 1980; Palmer *et al*. 1987; Taylor & Weston, 1988). The release of endothelium‐dependent relaxing factors is now acknowledged to occur in response to a wide range of vasoactive molecules (ACh, ATP, serotonin, histamine, bradykinin, substance P). However, although the signal transduction pathways for each of these vasoactive molecules are clearly present in the endothelium, the precise physiological source and role in the control of endothelial function of each remains to be definitely demonstrated (Sandow *et al*. 2012). This is particularly true of ACh. As a classical cholinergic neurotransmitter, ACh is released by nerve endings and is not normally expected to reach endothelial cells either through the vascular wall (Luscher & Vanhoutte, 1990; Taddei & Salvetti, 1997; Rees, 2002) or via blood (Vanhoutte, 1989). Yet, the endothelium is so exquisitely sensitive to ACh such that ACh is the most frequently used assay for both normal endothelial function and dysfunction in disease.

Notwithstanding the absence of a clear physiological role, the endothelium contains the enzymes necessary to synthesize, store and breakdown ACh (Parnavelas *et al*. 1985; Kirkpatrick *et al*. 2003). These include choline acetyltransferase (ChAT), the primary enzyme that catalyses ACh production, the vesicular ACh transporter (VAChT), which facilities storage of newly formed ACh in membrane vesicles, and acetylcholinesterase (AChE), which hydrolyses free ACh to form choline, acetate and water and terminate activity of the transmitter. In our studies examining the control of endothelial Ca^2+^ signalling, we observed a striking similarity between the complex multicellular signals that were initiated by exogenous ACh and those signals evoked by fluid flow. The similarity raised the possibility that endogenous ACh may underlie flow‐evoked endothelial responses.

The present study aimed to define the mechanisms of mechanical force (shear stress) transduction in the endothelium of intact arteries exposed to flow, and also to address whether local cholinergic mechanisms provide a mechanochemical transduction pathway responsible for promoting flow‐mediated dilatation. We show that endothelial organic cation transporters (OCTs) release ACh in response to mechanical activation by shear stress. The release of ACh requires mitochondrial generation of acetyl‐coenzyme A (acetyl‐CoA) and *de novo* ACh synthesis and non‐vesicular release via organic cation transporters. Interestingly, flow‐mediated ACh release is irreversibly inhibited by some brands of pentobarbital sodium that are used for animal dispatch. The data reveal that cholinergic signalling is a key element to endothelial mechanosensitivity, and the autocrine action of ACh explains vascular flow‐mediated dilatation.

## Methods

### Animals

All animal care and experimental procedure were carried out with the approval of the University of Strathclyde Local Ethical Review Panel [Schedule 1 procedure; Animals (Scientific Procedures) Act 1986, UK], under UK Home Office regulations. All experiments used either common carotid arteries or second‐order mesenteric arteries (as described) obtained from male Sprague–Dawley rats (10–12 weeks old; 250–350 g), killed by either (i) an overdose of CO_2_ or (ii) an overdose of pentobarbital sodium (200 mg kg^−1^, i.p.; Pentoject or Euthatal; Merial Animal Health Ltd, Woking, UK) as described.

### Flow‐mediated nitric oxide production

Nitric oxide production was assessed in the endothelium of *en face* carotid artery preparations, using a modification of a procedure for visualization of endothelial Ca^2+^ signalling (Wilson *et al*. 2016). Arteries were cut open along their longitudinal axis, using microscissors, and pinned out on a Sylgard block, with the lumen side upward. Arteries were then incubated with a loading solution consisting of the nitric oxide indicator, 4‐amino‐5‐methylamino‐2′,7′‐difluorofluorescein diacetate (DAF‐FM) (10 μm), 0.02% Pluronic F‐127 and 0.35% DMSO in physiological saline solution (PSS) for 60 min at room temperature. Following incubation, arteries were gently washed in PSS before the Sylgard blocks were placed face down on 0 grade thickness microscope coverslips fixed to the bottom of a custom bath chamber (length 3 cm, width 1.5 cm) and left for a further 60 min to allow intracellular de‐esterification of DAF‐FM. Stainless steel pins (diameter 200 μm) were used as spacers to ensure that the endothelium did not contact the coverslip, as well as to enable the endothelium to be exposed to fluid flow. With the Sylgard block in the chamber, laminar flow was provided by a syringe pump that was connected to the chamber via silicone tubing.

The dimensions of the flow chamber were 3 × 1.5 × 0.2 cm (length × width × height). The level of endothelial shear stress, *t*_w_ (dyne cm^−2^) was calculated (assuming a negligible arterial wall thickness) using the equation:

$$t_{w}=\frac{6\eta Q}{wh^{2}}$$

where *Q* is volumetric flow rate (cm^3 ^s^−1^) and η is the fluid viscosity (0.0089 dyne cm^−2^ for water). The endothelium was imaged using an inverted epi‐fluorescence microscope (TE2000U; Nikon, Tokyo, Japan). DAF‐FM was excited with 488 nm wide‐field epifluorescence illumination provided by a monochromator (Photon Technology International/Horiba UK, Ltd, Stanmore, UK) and fluorescence emission was imaged at 10 Hz using a 40× objective lens (numerical aperture 1.3), a 0.7× coupling lens and a back‐illuminated electron‐multiplying charge‐coupled device (EMCCD) camera (Cascade 512B; Photometrics, Tucson, AZ, USA) (1× binning). DAF‐FM fluorescence intensity measurements, averaged across the field‐of‐view, are expressed as baseline‐corrected fluorescence intensity (*F*/*F*_0_), where *F* is DAF‐FM fluorescence at time *t* and *F*_0_ is basal fluorescence intensity. Because nitric oxide does not dissociate from the dye, DAF‐FM *F*/*F*_0_ measurements represent the cumulative production of nitric oxide (Yi *et al*., 2002, 2005, 2011; Turovsky *et al*. 2013; Dolgacheva *et al*. 2016). To present the time course of nitric oxide production rate, we calculated discrete derivative [d(*F*/*F*_0_)/d*t*] traces. d(*F*/*F*_0_)/d*t* was obtained by convolving *F*/*F*_0_ traces with the first derivative of a Gaussian kernel in the programming language, Python. Discrete derivate [d(*F*/*F*_0_)/d*t*] signals are analogous to the first derivative of *F*/*F*_0_, if *F*/*F*_0_ was continuous in the time domain.

### Flow‐mediated endothelial Ca^2+^ signalling

Endothelial Ca^2+^ signalling was monitored in the endothelium of *en face* carotid artery and second‐order mesenteric artery preparations. The endothelium of *en face* preparations were incubated with a loading solution containing the fluorescent Ca^2+^ indicator, Cal‐520 acetoxymethyl ester (Cal‐520/AM) (5 μm), 0.02% Pluronic F‐127 and 0.35% DMSO in PSS for 30 min at 37 °C. Cal‐520/AM was used throughout as the indicator is reported to offer the highest signal‐to‐noise ratio of the most commonly available Ca^2+^ dyes (Lock *et al*. 2015). Indeed, in preliminary experiments (not shown), we found Cal‐520/AM to have a substantially increased dynamic range when compared to Oregon Green BAPTA‐1/AM. Following incubation, arteries were washed in PSS, and equilibrated at room temperature for 30 min before spontaneous or flow‐mediated Ca^2+^ signalling was monitored (10 Hz) as described above for nitric oxide production. In some flow experiments, a Nikon Ti‐S microscope with no coupling lens and an Andor iXON EMCCD (2X binning) camera were used. Following equilibration and confirmation that flow (1.5 ml min^−1^) elicited repeatable Ca^2+^ responses, flow‐mediated (1.5 ml min^−1^) endothelial Ca^2+^ responses were monitored before and after various treatments, as described in the text. Unless indicated otherwise, all treatments were tested after 20, 40 and 60 min of incubation. All summary data shown correspond to 20 min time point, unless otherwise stated. The results for all incubation times are summarized in Table 1.

**Table 1:** Effect of various pharmacological treatments on flow‐mediated Ca^2+^ signalling

	Peak Ca^2+^ response (peak *F*/*F*_0_)				Average Ca^2+^ response (average *F*/*F*_0_)			
Treatment	Control	Response 1	Response 2	Response 3	Control	Response 1	Response 2	Response 3
Ca^2+^‐free PSS (10 min intervals)	1.00 ± 0.00	0.50 ± 0.07*	0.28 ± 0.05*	0.04 ± 0.03*	1.00 ± 0.00	0.30 ± 0.06*	0.19 ± 0.09*	–0.03 ± 0.03*
SKF‐96365 (50 μm)	1.00 ± 0.00	0.41 ± 0.10*	0.42 ± 0.94*	0.39 ± 0.11*	1.00 ± 0.00	0.47 ± 0.1*	0.51 ± 0.12*	0.43 ± 0.18*
RuR (5 μm)	1.00 ± 0.00	1.02 ± 0.02	0.99 ± 0.01	1.02 ± 0.04	1.00 ± 0.00	1.03 ± 0.06	0.98 ± 0.10	1.00 ± 0.09
CPA (10 μm)	1.00 ± 0.00	0.16 ± 0.08*	0.07 ± 0.02*	0.20 ± 0.15*	1.00 ± 0.00	1.87 ± 0.57	1.50 ± 0.65	0.43 ± 0.98
2‐APB (100 μm)	1.00 ± 0.00	0.05 ± 0.01*	0.04 ± 0.01*	0.02 ± 0.0*	1.00 ± 0.00	0.03 ± 0.0*	–0.03 ± 0.0z*	–0.08 ± 0.0*
Ryanodine (30 μm)	1.00 ± 0.00	1.13 ± 0.04	1.10 ± 0.05	1.04 ± 0.01	1.00 ± 0.00	1.13 ± 0.03	1.40 ± 0.14	1.27 ± 0.06
U73122 (5 μm)	1.00 ± 0.00	0.00 ± 0.00*	0.00 ± 0.00*	0.00 ± 0.00*	1.00 ± 0.00	–0.05 ± 0.05*	–0.10 ± 0.03*	–0.05 ± 0.05*
Atropine (100 nm)	1.00 ± 0.00	0.00 ± 0.00*	0.00 ± 0.00*	–	1.00 ± 0.00	–0.08 ± 0.04*	–0.04 ± 0.01*	–
Acetylcholinesterase (4 U ml^−1^)	1.00 ± 0.00	0.06 ± 0.06*	–	–	1.00 ± 0.00	–0.14 ± 0.07*	–	–
TTX (10 μm)	1.00 ± 0.00	1.08 ± 0.08	1.12 ± 0.06	1.09 ± 0.09	1.00 ± 0.00	1.14 ± 0.16	1.12 ± 0.08	1.17 ± 0.12
Vesamicol (10 μm)	1.00 ± 0.00	1.03 ± 0.03	1.08 ± 0.06	1.09 ± 0.05	1.00 ± 0.00	1.13 ± 0.02	1.19 ± 0.08	1.18 ± 0.07
BromoACh (50 μm)	1.00 ± 0.00	0.52 ± 0.24*	0.48 ± 0.18*	0.61 ± 0.13*	1.00 ± 0.00	0.32 ± 0.16*	0.32 ± 0.07*	0.51 ± 0.11*
CCCP (5 μm)	1.00 ± 0.00	0.00 ± 0.05*	–	–	1.00 ± 0.00	–0.06 ± 0.02*	–	–
–Gluc/+SP PSS	1.00 ± 0.00	1.29 ± 0.38	–	–	1.00 ± 0.00	1.19 ± 0.33	–	–
+Gluc/–SP PSS	1.00 ± 0.00	0.76 ± 0.09*	–	–	1.00 ± 0.00	0.631 ± 0.090	–	–
–Gluc/–SP PSS	1.00 ± 0.00	0.49 ± 0.13*	–	–	1.00 ± 0.00	0.409 ± 0.141	–	–
Corticosterone (100 μm)	1.00 ± 0.00	0.66 ± 0.12*	0.59 ± 0.18*	0.55 ± 0.19*	1.00 ± 0.00	0.561 ± 0.116	0.497 ± 0.178	0.418 ± 0.140
Decynium 22 (1 μm)	1.00 ± 0.00	0.02 ± 0.02*	–	–	1.00 ± 0.00	–0.03 ± 0.08*	–	–
High‐K^+^ PSS	1.00 ± 0.00	0.02 ± 0.01*	0.09 ± 0.04*	0.06 ± 0.01*	1.00 ± 0.00	–0.04 ± 0.02*	–0.02 ± 0.04*	0.01 ± 0.03*
Barium (1 mm)	1.00 ± 0.00	0.82 ± 0.10	0.84 ± 0.11	0.91 ± 0.11	1.00 ± 0.00	0.63 ± 0.16	0.60 ± 0.16	0.83 ± 0.14
DIDS (10 μm)	1.00 ± 0.00	1.12 ± 0.04	1.11 ± 0.05	1.14 ± 0.15	1.00 ± 0.00	1.09 ± 0.07	1.04 ± 0.10	1.08 ± 0.12
CFTR_inh_172 (20 μm)	1.00 ± 0.00	0.97 ± 0.09	0.94 ± 0.02	0.86 ± 0.04	1.00 ± 0.00	0.95 ± 0.18	0.87 ± 0.05	0.89 ± 0.073
Apyrasae (4 U ml^−1^)	1.00 ± 0.00	0.93 ± 0.15	1.00 ± 0.11	0.98 ± 0.13	1.00 ± 0.00	1.03 ± 0.19	1.06 ± 0.18	0.99 ± 0.19
Suramin (100 μm)	1.00 ± 0.00	1.09 ± 0.03	1.00 ± 0.03	0.96 ± 0.02	1.00 ± 0.00	1.11 ± 0.04	1.02 ± 0.03	1.01 ± 0.03
Probenecid (250 μm)	1.00 ± 0.00	1.08 ± 0.03	1.09 ± 0.05	1.09 ± 0.05	1.00 ± 0.00	1.14 ± 0.04	1.18 ± 0.03	1.16 ± 0.07

Results are the mean ± SEM. Treatment with Ca^2+^‐free PSS was tested at 10 min intervals. All other treatments were tested at 20 min intervals following incubation. ^*^ *P* < 0.01.

### Photolysis of caged inositol trisphosphate (IP_3_)

In some experiments, endothelial Ca^2+^ signalling was examined in response to local photolysis of caged IP_3_. The endothelium of e*n face* arteries was first loaded with Cal‐520/AM (5 μm), as described above, and then incubated with a second loading solution containing a membrane permeant caged IP_3_, caged IP_3_ 4,5‐dimethoxy‐2‐nitrobenzyl (10 μm), 0.02% Pluronic F‐127 and 0.35% DMSO in PSS for 30 min at 37 °C. Photolysis of caged IP_3_ was achieved using a frequency tripled neodymium: yttrium aluminium garnet (Nd:Yag; wavelength 355 nm) laser (Rapp Optoelektronic, Hamburg, Germany) attached directly to the TE2000U microscope system (McCarron *et al*. 2010). The position of the photolysis site (∼2 μm diameter) and the irradiation time (1 ms) were computer controlled (Rapp Optoelektronic) and images were recorded at 10 Hz. Identical UV flashes in the absence of caged IP_3_ evoked no detectable Ca^2+^ response.

### Endothelial mitochondria

Ca^2+^ signalling and mitochondria were visualized simultaneously, as described previously (Chalmers & McCarron, 2008; Olson *et al*. 2012). In brief, tetramethylrhodamine ethyl ester (TMRE) (120 nm) was added to the MOPS perfusion solution (MOPS PSS) and the endothelium was incubated 10 min. Subsequently, TMRE (120 nm) was present in all perfusion solutions. Ca^2+^ and TMRE images were acquired sequentially using the TE2000U microscope system described above equipped with a 100× objective (numerical aperture 1.4). The exposure on each channel was 50 ms, resulting in an acquisition rate of 10 Hz for each channel. Minimal photobleaching of TMRE was observed over the 5 min recording periods used.

### Ca^2+^ signal analysis

**Figure 1:**
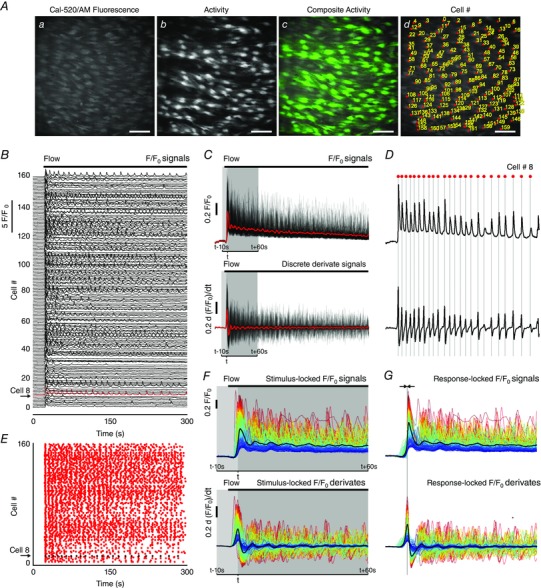
Large‐scale imaging and analysis of Ca^2+^ signalling in the intact endothelium *A*, Ca^2+^ images of intact endothelium: (*a*) averaged fluorescence intensity showing endothelial cells; (*b*) flow‐evoked Ca^2+^ activity, visualized by taking the standard deviation of the sequential subtraction image data; (*c*) composite overlay of (a) (grey) and (b) (green); (*d*) Ca^2+^ image with each cell identified and numbered (‘Cell #’). All image scale bars = 50 μm. *B*, 5 min long Ca^2+^ traces for each of the 160 cells identified in (*A*). The traces have been baseline‐corrected (*F*/*F*_0_). Stimulation with fluid flow is indicated by the black bar and individual traces have been offset along the *y*‐axis. Numbers along the left side correspond to the Cell # in (*Ad*). *C*, *F*/*F*_0_ Ca^2+^ traces (top) shown in (*B*) overlaid on top of each other, and derivate Ca^2+^ [d(*F*/*F*_0_)/d*t*] traces (bottom) overlaid on top of each other. The average is overlaid in red. *D*, as in (*C*) (same scales) but for a single cell (Cell # 8 shown in *B*). A ‘zero crossing’ detector was used to automatically identify the Ca^2+^ transient peaks from the derivative data. Peaks are indicated by the grey lines and red dots above the trace. *E*, rastergram display of Ca^2+^ activity: the *x*‐axis is time, whereas the *y*‐axis is the Cell # identified in (*Aiv*) and (*B*). A dot in the rastergram indicates that a peak in the Ca^2+^ signal of the indicated cell occurred at time, *t*. Cell # 8 is again highlighted. *F*, ‘stimulus‐locked’ *F*/*F*_0_ Ca^2+^ traces (top) and derivate Ca^2+^ [d(*F*/*F*_0_)/d*t*] traces (bottom) from data shown in (*C*). Stimulus‐locked Ca^2+^ signals are traces extracted with respect to the average time (*t*; 10 s preceding and 60 s following) at which the first peak, arising after the start of the stimulus, in each derivate trace occurs (i.e. signals are locked to the stimulus). The average is overlaid (thick black line). *G*, ‘response‐locked’ *F*/*F*_0_ Ca^2+^ traces. Response‐locked Ca^2+^ signals are aligned in time with respect to the time at which the first Ca^2+^ peak has occurred after the start of the stimulus. This alignment facilitates extraction of signalling metrics. Derivate Ca^2+^ [d(*F*/*F*_0_)/d*t*] traces (bottom) from data shown in (*C*) and (*F*) (same scales as *F*), which were used to facilitate alignment (response‐locking) signals. *F* and *G*, traces are coloured according to the magnitude of first peak in each cellular response (red, largest; blue, smallest).

Ca^2+^ signalling was imaged in large fields (∼150 cells) of intact endothelium with high spatial resolution (Fig. 1 *Aa*). To facilitate visual inspection of endothelial Ca^2+^ signals in all cells, image stacks were generated to show propagating Ca^2+^ wavefronts. These Ca^2+^ wavefront image stacks were generated by calculating the forward difference of changes in fluorescence intensity (*F_t_* − *F_t_*_−1,_ obtained by sequential subtraction) (Bradley *et al*. 2003; McCarron *et al*. 2010). The propagating Ca^2+^ wavefront stacks were then converted to single images that showed all cells that exhibited Ca^2+^ activity. This single image was created by taking the standard deviation (STDev) of intensity of the sequential subtraction image stacks (Fig. 1 *Ab*). Throughout the present study, STDev images are presented overlaid on average‐intensity projections to create colour composites showing Ca^2+^ activity (Fig. 1 *Ac*).

### WAVE (whole‐cell average) Ca^2+^ signal analysis of flow‐evoked activity

As reported in the Results, fluid flow evoked whole‐cell propagating Ca^2+^ waves. The whole‐cell, or ‘global’ signals are similar to those seen when the endothelium is activated by exogenous ACh. We previously developed an automated analysis routine for analysing these signals. This procedure enables rapid extraction of Ca^2+^ signals and quantification of signalling metrics for every cell in the field‐of‐view and, furthermore, enables the response of individual cells to be matched throughout lengthy experimental protocols (Wilson *et al*., 2015, 2016). In the present study, the large number of cells visualized rendered manual analysis impractical and so we used a modified version of this largely automated data processing procedure. This algorithm extracts Ca^2+^ signals by averaging fluorescence intensity within a whole‐cell region of interest (ROI) and thus enables the quantification of whole‐cell Ca^2+^ signalling metrics. The action of the algorithm, which we now name WAVE (Whole‐cell AVErage) Ca^2+^ signal analysis, is: (1) all individual cells within the field‐of‐view are identified and, for each cell, a ROI that encompasses the majority of the cell area is created; (2) time‐dependent fluorescence intensities (Ca^2+^ signal) are extracted from each ROI; (3) each Ca^2+^ signal is normalized to its corresponding baseline; (4) Ca^2+^ signalling metrics, such as peak amplitudes and oscillation frequency, are rapidly and objectively determined for all cells; and (5) Ca^2+^ signals and summary data are presented in pictorial form.

#### Whole‐cell ROI generation

To enable ROIs to be generated by intensity thresholding, we first created average‐intensity projections of each image stack (Fig. 1 *Aa*). Sharpened images were obtained by applying an unsharp mask filter. ROIs were then generated by applying a threshold to intensity levels. The resulting ROIs encompassed the majority of the area of each cell. These whole‐cell ROIs were verified and any erroneous ROIs were corrected manually. Our longitudinal experimental design required a comparison of Ca^2+^ responses before and after pharmacological intervention (at multiple time‐points) in the same cells. Because of the incubation/equilibration periods, these data were acquired across multiple recordings. Occasionally, the microscope field‐of‐view drifted slightly between acquisitions, and so ROI sets were aligned, across separate image acquisitions, using an automated alignment plug‐in in FIJI (Tseng *et al*. 2011). Thus, unless otherwise indicated (as in subcellular analysis), each ROI contained a single cell and ROIs generated for control data were applied to response (e.g. after drug treatment) data. Each ROI (cell) was assigned an identification number, termed ‘Cell #’, to permit direct comparison (pairing) of responses from precisely the same individual cells under control conditions and after pharmacological intervention (Fig. 1 *Ad*).

#### Automated whole‐cell Ca^2+^ signal extraction

Time‐dependent whole‐cell Ca^2+^ signals were extracted from the fluorescence intensity (*F*) within each of the whole‐cell ROIs for each frame of the image recordings (Wilson *et al*., 2015, 2016). Raw fluorescence signals were then expressed as baseline‐corrected fluorescence intensity (*F*/*F*_0_) (Fig. 1 *B*–*D*, top) by dividing each fluorescence intensity trace by the average value of a 50‐frame (5 s) baseline‐period (*F*_0_) at the start of each trace. *F*_0_ was determined for each cell. *F*/*F*_0_ traces were then smoothed using 11‐point (1.1 s), third‐order polynomial Savitzky–Golay filter.

#### Automated whole‐cell Ca^2+^ signal analysis

The time of occurrence and amplitude of peaks in each whole‐cell Ca^2+^ (*F*/*F*_0_) signal (Fig. 1 *B*) were determined using an automated computer algorithm. First, for each whole‐cell Ca^2+^ signal, we calculated discrete (first) derivative Ca^2+^ signals [d(*F*/*F*_0_)/d*t*] (Fig. 1 *C* and *D*, bottom), as described for nitric oxide measurements above. These derivate signals can be used to infer Ca^2+^ activity in the corresponding *F*/*F*_0_ signal (Smetters *et al*. 1999) because an increase or decrease in *F*/*F*_0_ corresponds to a positive or negative deflection in the discrete derivative, respectively. At the peak (or nadir) of a spike in a *F*/*F*_0_ Ca^2+^ signal, the derivative changes sign from positive to negative (or negative to positive). Thus, we used a ‘zero‐crossing detector’ to identify the times at which the sign of each derivate signal changed from positive to negative or negative to positive. The zero‐crossing detector identifies all times of ‘zero‐crossing’ (all times at which each derivate signal equals zero). For each whole‐cell Ca^2+^ signal, these times were organized into sequential pairs. The magnitude of the critical point (peak or nadir) between each pair was then extracted. Any peak or nadir in the derivate trace with a magnitude less than three times the standard deviation of baseline noise (5 s of each derivate trace) was discarded; three times the standard deviation was considered to be the threshold. The sign of each critical point that was greater in magnitude than the threshold level of the derivate signal was used to determine whether the ‘zero‐crossing’ pair corresponded to a detectable rise or fall in the *F*/*F*_0_ signal. Thus, the zero‐crossing detector enabled the times of all peaks (above threshold) in the derivate signal to be extracted. These peaks correspond to the rising edge in the *F*/*F*_0_ signal. The time at which the peak occurred in the *F*/*F*_0_ signal was then extracted by measuring the maximum *F*/*F*_0_ value in a 5 s window following the identified rising edge. The occurrence of any detectable peak in each trace was used to determine whether the corresponding cell had responded. If cells responded, they were defined as ‘Active’. The times of occurrence of the first peak in each Ca^2+^ response were used to generate latency profiles of cellular Ca^2+^ responses. These latency profiles are presented as histograms with time *t* = 0 corresponding to the time of the first detected peak (i.e. the time of the first peak in the signal of the first responding cell). The times of occurrence of each peak in the Ca^2+^ data were also used to generate rastergrams of Ca^2+^ activity (Fig. 1 *E*). The rastergram plots show peaks in the Ca^2+^ signals as dots at the time of their occurrence. These plots provide a convenient display of the oscillatory activity of many cells and permit visual identification of cells that respond to various stimuli. The times were also used to extract conventional measurements from the corresponding *F*/*F*_0_ data as described below.

The endothelial preparations used in the present study exhibited minimal photobleaching, or response ‘run‐down’, and remained in focus for extended periods of time (>10 min) permitting repeated imaging of the same large field of intact endothelia over several hours. Routine recordings typically consisted of imaging periods of ∼2 min performed every 20 min of the duration of each experiment. A typical experimental protocol consisted of two control recordings (each 2 min long and recorded at 20 min intervals). These two control recordings were followed by another recording of 5 min in duration, during which an initial control response was obtained and a pharmacological intervention was performed (e.g. addition of a pharmacological inhibitor). This permitted us to record the effects drugs during their addition. After treatment, we then recorded another three responses (each 2 min in duration and recorded at 20 min intervals).

To present the Ca^2+^ data in a convenient pictorial form, *F*/*F*_0_ signals are shown with respect to the time at which flow was initiated (typically 10 s before and 60 s after the stimulus) (Fig. 1 *F*). These *F*/*F*_0_ signals are displayed ‘locked’ to the time of stimulus and termed ‘stimulus‐locked’. However, there was a considerable spread in the time at which each cell responded to stimuli. Therefore, we automatically aligned the *F*/*F*_0_ traces based on the time at which the first peak occurred in each and every cell (Fig. 1 *G*). Thus, these aligned *F*/*F*_0_ signals are ‘locked’ to the first response of each cell (typically 10 s before and 60 s after the peak) and are termed ‘response‐locked’ (Fig. 1 *F*). In cases where no peak was identified in a given trace, those Ca^2+^ signals were instead extracted with respect to the average time at which all other cells responded. Alignment of the signals in this way enabled Ca^2+^ signalling metrics to be extracted with ease. Measurements extracted from the aligned signals included the the amplitude of the first peak (peak Δ*F*/*F*_0_), the average signal level during the first 60 s following the first peak (average Δ*F*/*F*_0_) and the inverse of the number of peaks occurring within this 60 s period (i.e. oscillation frequency). Because all signals were aligned so that the initial peak of each coincided in time (typically *t* = 10 s), peak amplitudes (peak Δ*F*/*F*_0_) were calculated by measuring the values of each response‐locked signal at this time. In signals where no peak was identified, peak Δ*F*/*F*_0_ was taken to be zero. Thus, our measure of peak Ca^2+^ response, as shown in summary data (normalized to control responses) throughout the present study, accounts for the Ca^2+^ response integrated across all cells within the field‐of‐view. Time‐average signal levels were calculated by taking the average of the 60 s period of all signals following this time. Furthermore, the frequency (events cell^–1^ min^−1^) of each active whole‐cell Ca^2+^ signal was calculated by taking inverse of the number of peaks occurring within this same 60 s period. Because of possible errors arising from signals falling below baseline values, area under the curve measurements were not calculated.

Representative whole‐cell Ca^2+^ traces from all cells within a field‐of‐view are presented throughout the present study as composite plots where the colour of each trace (Fig. 1 *F* and *G*) represents the amplitude of the initial peak in the response to flow. A full heat map range was used to colour the traces (blue, small flow response; red, large flow response. The colour‐coding was maintained throughout each series of experiments on a single endothelial preparation (i.e. the colour assigned to each cell is calculated from control responses and preserved across datasets). In scatter plots showing the (initial) peak Ca^2+^ responses or time‐averaged Ca^2+^ responses, datapoints are colour‐coded according to the plotting density of individual points and pairing of responses are indicated by the connecting lines. Linescan kymograph images were generated using the ‘reslice’ function in FIJI. These images were generated by measuring fluorescence across a specified single (or multipoint) line placed on raw image stacks, and their evolution over time is displayed as a pseudocoloured representation. 3‐D surface plots were generated using custom macros employing the ‘3D_Surface_Plot’ function in FIJI. In some images, ‘full‐field’ average responses are shown. Full‐field average responses are derived from a single ROI encompassing the entire field‐of‐view.

A release of the source code for our WAVE Ca^2+^ signal analysis algorithm, together with sample experimental data, is currently under preparation. This will be freely available via the University of Strathclyde data repository, ‘KnowledgeBase’ (https://pure.strath.ac.uk/portal/en/datasets). In the meantime, readers are encouraged to contact the authors if they would like to share the current tools described above.

### Spontaneous Ca^2+^ signal analysis

The stability of our experimental apparatus (i.e. lack of focus drift, stage drift) and lack of smooth muscle tone/rhymicity enabled recordings of endothelial Ca^2+^ activity for extended periods of time. We recorded basal endothelial Ca^2+^ activity in carotid and mesenteric endothelial preparations for periods of 60 s. The occurrence of spontaneous Ca^2+^ activity in these recordings was first confirmed by visual inspection and by placing subcellular regions of interest within the boundaries of cells exhibiting spontaneous activity. Rigorous manual analysis of these data was impractical as a result of the large number of cells visualized. As such, we performed an analysis of this data using the WAVE Ca^2+^ signal analysis algorithm described above. However, as described in the Results, WAVE does not assess the spatial profile of extracted Ca^2+^ signals and may miss very small‐amplitude subcellular events, and so may not be well‐suited to the analysis of highly localized spontaneous Ca^2+^ signals. Instead, we used an alternative algorithm, developed by an independent group specifically for the automated detection and analysis of localized Ca^2+^ signals in camera‐based imaging data (Ellefsen *et al*. 2014). This algorithm, and its use in assessing and characterizing localized Ca^2+^ signalling in a number of cell types, has been demonstrated (Ellefsen *et al*. 2014; Lock *et al*. 2015, 2016; Schmunk *et al*. 2015). Nevertheless, below we include a brief description of our use of the algorithm.

#### Image pre‐processing and event detection

Raw image stacks from recordings of spontaneous activity were first processed by dividing each frame in the stack by the mean of all frames and subtracting a value of 1 from every pixel. The resulting fluorescence of each pixel thus represents a ratio (Δ*F*/*F*_AVG_) of the increase in fluorescence (Δ*F*) of that pixel relative to its mean fluorescence (*F*_AVG_) throughout the recording. The standard deviation of the resulting image stack was then normalized to 1 by dividing each frame in the stack by the standard deviation of all frames, and the resultant stack further processed by applying a Guassian blur (two pixel radius). These image stacks were then converted into binary form by applying a threshold. The resulting binary representation is thus a matrix where a pixel value of 1 (or 0) indicates the presence (or absence) of a Ca^2+^ event above threshold. All image pre‐processing was performed in FLIKA, an interactive image processing suite written in Python (http://flika-org.github.io/).

#### Event analysis

Spontaneous Ca^2+^ event data were analysed using the ‘detect puffs’ plugin of FLIKA for automated analysis of spontaneous signals. The plug‐in extracts the 3‐D (*x*, *y*, *t*) co‐ordinates that encompass each Ca^2+^ event, identified in binary Δ*F*/*F*_AVG_ image stacks. Unique Ca^2+^ events are first identified using a clustering algorithm based on the premise that a pixel corresponding to a cluster centre may be recognized as a local density maxima with a relatively large distance from points with higher densities (Rodriguez & Laio, 2014). Once identified, the co‐ordinates of a box surrounding each cluster are mapped onto the normalized Δ*F*/*F*_AVG_ image stack, extended (*xy* padding) by a user‐defined number of pixels in the *xy* planes, and a mean spatial image of each event is created by averaging each pixel intensity within the time window. These images are then normalized to the highest pixel value, and a 2‐D elliptical Gaussian function is fitted to this mean spatial image. The Gaussian fitting function reports the *x* and *y* centroid positions, *x* and *y* standard deviations, and angle of the long axis of the resulting elliptical function. Ca^2+^ event traces are then extracted from the Δ*F*/*F*_AVG_ image stack by averaging pixel values within square ROIs of user‐defined width centered around each of the centroids calculated by the Gaussian fitting process for a user‐defined number of frames preceding and following event. Once these event traces are extracted, the maximum amplitude of the event is calculated automatically. Because multiple events may arise from the same site, detected events that occur within a user‐defined distance from each other are grouped together and considered to arise from a single site. The centroid location of each of these sites is calculated by taking the mean (unweighted) of a square that encompasses the centroids of all the grouped events. Ca^2+^ traces for each site are then extracted by averaging fluorescence intensity within a square ROI centered on each group centroid. In the present study, the settings used were: ROI width = 5 pixels (∼2.9 um); number of frames following/preceding event = 25, *xy* padding = 40 pixels (∼23 um), group radius = 15 pixels (∼8.5 um) that occurred within a 20 pixel (∼11.5 um) radius were grouped and considered to be arising from the same site. The results are presented as peak event amplitude (Δ*F*/*F*_AVG_), spatial spread (μm^2^), frequency of Ca^2+^ events per site (Hz; events s^−1^) and the fraction of cells exhibiting sites of spontaneous Ca^2+^ events. The spatial spread of each event was determined by calculating the elliptical area under the fitted 2‐D Gaussian.

In some experiments, the sites of spontaneous basal Ca^2+^ events and flow‐evoked Ca^2+^ waves were examined in the same artery. In these experiments, basal activity was recorded after an equilibration period (no flow) of 20 min. Flow‐evoked Ca^2+^ activity was then recorded in the same field of endothelial cells. Single images showing regions of spontaneous activity were created by generating STDev images of the corresponding sequential subtraction image stack of the basal recording. Similarly, single images indicating the sites of origin of flow‐evoked Ca^2+^ waves were created by generating STDev images of a 2 s period (20 frames) of the sequential subtraction image stack immediately following the response to flow. STDev images were converted to binary form using Huang's method for thresholding in FIJI, and the extent of co‐localization was assessed by calculating the amount of area that overlapped for each set of binary STDev images.

### Flow‐mediated dilatation

Flow‐mediated dilatation of pressurized intact rat carotid arteries was examined as described previously (Craig & Martin, 2012). In brief, segments (∼20 mm in length) of carotid arteries were dissected and cleaned of adhering fat and connective tissue. Arteries were mounted onto two stainless steel cannula (21 G) in a pressure myography bath (11OP; Danish Myo Technology A/S, Aarhus, Denmark) filled with PSS and secured with nylon suture thread. Arteries were flushed with PSS to clear blood from the lumen. The myography bath was mounted on an inverted microscope and the temperature of the bath brought to 37 °C. The myograph cannulae were connected to a perfusion system that enabled adjustment of lumenal flow rate and intraluminal pressure. Lumenal flow was provided by a peristaltic pump (Minipuls 3; Gilson Scientific Ltd, Luton, UK) connected to the proximal cannula via silicone rubber tubing and a heat exchange coil that warmed perfusion solutions to 37°C. A custom, inline pulse dampener was employed to reduce fluctuations in flow induced by the peristaltic pump. A silicone rubber outflow tube, connected to the distal cannula, and leading to a waste reservoir enabled variable intraluminal pressure: increasing/decreasing the height of the reservoir enabled pressure to be controlled. Once mounted, arteries were pressurized, by perfusing PSS through the system at a speed of 0.1 ml min^−1^ and raising the height of the waste reservoir, until a pressure of 120 mmHg was reached. Any buckle resulting from reapplication of pressure was then removed by straightening the vessel and arteries were equilibrated at 37 °C for 30 min. The carotid artery exhibits minimal spontaneous tone (Craig & Martin, 2012; Wilson *et al*. 2016). Thus, after equilibration, arteries were pre‐constricted with phenylephrine (1 μm) and then assessed for endothelial viability by extraluminal application of ACh (100 μm). Note that ACh is less effective applied extralumenally than when applied directly to the endothelium of a large artery, such as the rat carotid artery. Indeed, in a previous study of endothelial Ca^2+^ imaging, we found the EC_50_ for ACh to be 1000‐fold lower in *en face* carotid artery preparations, in which ACh had free access to the endothelium, than for pressurized carotid artery preparations, in which ACh had to traverse the vascular wall (Wilson *et al*. 2016). The thick vessel wall presumably acts as a barrier to diffusion. Similar findings have been reported previously. For example, the potency of extraluminally applied ACh was reported to be ∼1:50 of intraluminally applied ACh in the dog mesenteric artery (Toda *et al*. 1990) and 50–100 times less potent in femoral artery (Angus *et al*. 1983; Toda *et al*. 1988). Bradykinin, when applied extraluminally, is unable to evoke any relaxant responses in isolated porcine coronary arteries (independent of enzymatic degradation and luminal pressure) but is able to evoke responses when applied intraluminally (Tankó *et al*. 1999).

All pre‐constricted arteries exhibited >50% dilatation to ACh and were thus considered viable. After confirmation of viability, flow‐mediated dilatation (2 ml min^−1^ lumenal flow) was assessed in the pre‐constricted arteries. In all experiments, arteries were imaged using a 2.5× objective and a charge‐coupled‐device camera, and pressure was monitored using two pressure transducers contained within the pressure myograph. Data were streamed to a computer, and arterial diameter was measured using online video dimension analyser software (MyoVIEW; Danish Myo Technology A/S).

### Drugs and solutions

SKF‐96365, U73122, vesamicol, corticosterone and cystic fibrosis transmembrane regulator inhibitor 172 (CFTR_inh_172) were obtained from Tocris (St Louis, MO, USA). Cal‐520/AM and TTX were obtained from Abcam (Cambridge, MA, USA). Pluronic F‐127 was obtained from Invitrogen (Carlsbad, CA, USA). All other drugs and chemicals were obtained from Sigma (St Louis, MO, USA). The PSS consisted of (mm): 145 NaCL, 4.7 KCl, 2.0 MOPS, 1.2 NaH_2_PO_4_, 5.0 glucose, 2.0 pyruvate, 0.02 EDTA, 1.17 MgCl_2_, 2.0 CaCl_2_, adjusted to pH 7.4 with NaOH. High‐K^+^ PSS contained 70 mm KCl, which replaced NaCl on an equimolar basis. All solutions were freshly prepared each day and pyruvate (S8636) was replaced on a weekly basis.

### Statistical analysis

Summary data are presented graphically, as averaged, paired responses from each of *n* arteries obtained from *n* different animals (biological replicates). The mean ± SEM is reported for the *n* biological replicates. In some cases, the total number of cells from which averaged measurements were made is reported as *N* technical replicates. Apart from experiments performed in High‐K^+^ PSS, the Ca^2+^ responses of the same individual cells were paired. In those experiments using High‐K^+^ PSS, arteries contracted significantly and, although there was some overlap in the cells imaged, pairing individual cells was not possible. Unless indicated otherwise, all values were normalized to control responses. Responses were analysed statistically using one‐way ANOVA with Dunnet's *post hoc* test, as appropriate. All statistical analyses were performed using Prism, version 6.0 (GraphPad Software, La Jolla, CA, USA). *P* < 0.01 was considered statistically significant.

## Results

### Flow‐mediated dilatation of carotid arteries is mediated by nitric oxide

**Figure 2:**
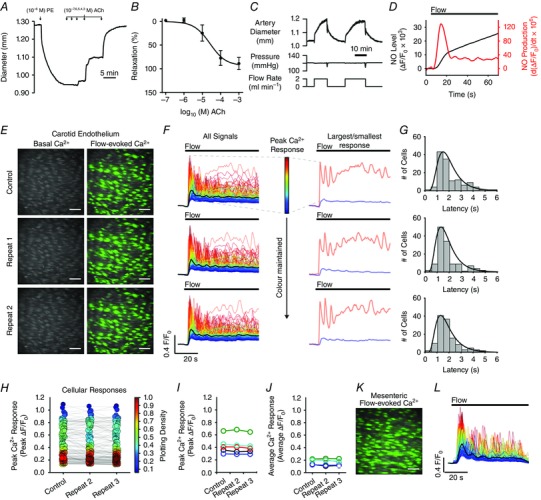
Flow‐mediated endothelial responses in carotid and mesenteric arteries *A* and *B*, representative trace (*A*) and summary data (*B*) (EC_50_ = 26.2 ± μm, 95% confidence interval, 16.9–49.6 μm; *n* = 6) of ACh‐mediated relaxation of pre‐constricted (1 μm phenylephrine) pressurized (120 mmHg) rat carotid arteries. *C*, representative trace of flow‐induced (2 ml min^−1^) vasodilatation of a pre‐constricted pressurized carotid artery. *D*, a typical recording of DAF‐FM fluorescence intensity (*F*/*F*_0_, black line) and calculated nitric oxide production [d(*F*/*F*_0_)/d*t*; red line] obtained from an *en face* endothelium preparation stimulated by fluid flow. *E*, representative endothelial Ca^2+^ images of of an *en face* carotid artery preparation before (left) and during (right) stimulation with fluid flow (1.5 ml min^−1^) on three consecutive occasions. *F*, whole‐cell Ca^2+^ signals from images shown in (*E*). Ca^2+^ traces are coloured according to the magnitude of the peak response (the colour assigned to each cell is preserved across each acquisition, and the average is overlaid in black). Right: responses of the two cells exhibiting the largest and smallest response to flow. *G*, histograms illustrating the temporal spread of time to first peak in Ca^2+^ responses (latency). *H*, summary data from the single experiment shown in (*E*) to (*G*). Data points are coloured according to the density of plotted points. *I* and *J*, paired summary data illustrating changes in peak (*I*) and time‐averaged (*J*) Ca^2+^ response values, averaged across individual cells per experiment (*n* = 5). *K*, Ca^2+^ image of the endothelium of an *en face* mesenteric artery during (right) stimulation with fluid flow (1.5 ml min^−1^). *L*, whole‐cell, colour‐coded Ca^2+^ signals from experiment shown in (*K*). All image scale bars = 50 μm.

To examine the physiological relevance of endothelial mechanotransduction, ACh‐ and flow‐mediated dilatations were studied in pressurized rat carotid arteries mounted in a pressure/flow myograph. Pre‐constricted arteries (1 μm phenylephrine) dilated to exogenous ACh in a concentration‐dependent manner (Fig. 2 *A* and *B*) and responded to the induction of flow (2 ml min^−1^; ∼2.5 dyne cm^–2^) with a rapid dilatation that persisted until flow was stopped (Fig. 2 *C*). The maximal relaxation to flow was 32 ± 6% (*n* = 5). Flow‐mediated dilatation of rat carotid arteries is dependent on an intact endothelium and is attenuated by blockade of nitric oxide synthase using l‐NAME (Martin *et al*. 1996; Bergaya *et al*. 2001; Craig & Martin, 2012). To confirm the involvement of nitric oxide in flow‐mediated dilatation, we visualized nitric oxide production in the endothelium of cut‐open arteries (*en face* preparation) loaded with the fluorescent indicator, DAF‐FM. Figure 2 *D* (black line) shows a representative trace of DAF‐FM fluorescence intensity from an experiment in which the endothelium was stimulated by fluid flow (1.5 ml min^−1^). Because nitric oxide irreversibly binds to DAF‐FM, measured fluorescence intensities represent the total accumulation of nitric oxide. To show the time‐dependent changes in nitric oxide production, we calculated derivate DAF‐FM fluorescence signals (Yi *et al*. 2002). The differential calculation (Fig. 2 *D*, red line) illustrates that the onset of flow induces a rapid increase in nitric oxide generation, whereas steady flow maintains sustained nitric oxide production.

### Flow‐mediated endothelial Ca^2+^ signalling

The generation of nitric oxide is a Ca^2+^‐dependent process (Falcone *et al*. 1993). To test whether fluid flow stimulated endothelial Ca^2+^ signalling, we imaged endothelial cells of intact carotid arteries loaded with the fluorescence Ca^2+^ indicator, Cal‐520/AM (Fig. 2 *E*). Flow (1.5 ml min^−1^, ∼2.5 dyne cm^−1^) caused a rapid rise in [Ca^2+^]_i_ in all cells across the field‐of‐view (∼150 cells per experiment) (Fig. 2 *F–H*; see also Supporting information, Movie S1). As measured from whole‐cell ROIs, the rise in [Ca^2+^]_i_ of individual cells was heterogeneous and oscillatory (Fig. 2 *E–I*; see also Supporting information, Movie S1) and arose from the wave‐like propagation of whole‐cell Ca^2+^ waves within individual cells and throughout clusters of endothelial cells. The frequency of whole‐cell Ca^2+^ waves ranged from 0.017 to 0.267 Hz (i.e. all 751 endothelial cells, imaged in five separate experiments, exhibited one to 16 peaks in the 60 s period of the Ca^2+^ response following activation). The frequency, averaged across individual experiments, was 0.106 ± 0.020 Hz (*n* = 5). Because of the apparent multicellular nature of these waves, the endothelial Ca^2+^ response was spatiotemporally complex (see Supporting information, Movie S1). However, when the responses of each cell were matched to itself across three separate fluid flow activations (Fig. 2 *H* and *I*), the Ca^2+^ responses measured each time were approximately reproducible (*n* = 5) (Fig. 2 *E–J*).

**Figure 3:**
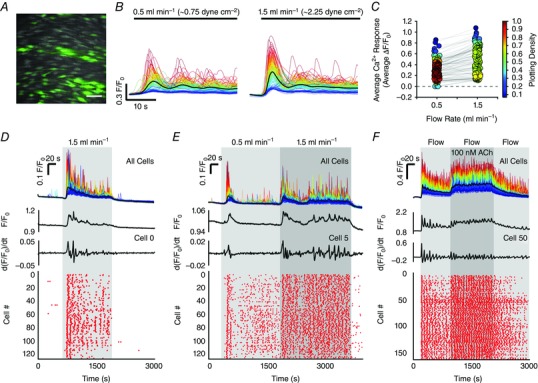
Flow‐induced endothelial Ca^2+^ signalling dynamics *A*, fluorescence Ca^2+^ image of carotid artery endothelium during flow. Scale bar = 50 μm. *B*, whole‐cell Ca^2+^ signals, from endothelial cells shown in (*A*), arising from the flow rates indicated above the trace. The average response is overlaid in black (*C*) Ca^2+^ measurements (peak Δ*F*/*F*_0_) paired from the same cells at flow rates of 0.5 and 1.5 ml min^−1^ from the data shown in (*B*). *D*–*F*, Ca^2+^ dynamics in populations of endothelial cells exposed to various flow rates and exogenous ACh. From top to bottom: whole‐cell Ca^2+^ responses obtained from all cells across the field‐of‐view; a Ca^2+^ trace; a corresponding derivate Ca^2+^ trace from a single cell; and a rastergram display of Ca^2+^ activity. In (*D*), the endothelium was exposed to a single stepwise increase in flow rate (periods of flow indicated by the grey box). In (*E*), serial stepped increases in flow rate were applied. In (*F*), ACh (100 nm) was added to the perfusion solution after flow‐evoked Ca^2+^ signalling had already been established (data shown in the Supporting information, Movie S3). The traces (*F*, top) are sorted and colour‐coded based on amplitude of initial response to flow (blue, small flow response; red, large flow response). The colour applied to the cell is maintained right through the experiment. Thus, the cells retain the same colour applied to them after ACh (i.e. those cells that were red coloured are precisely the same cells that were coloured red for flow; those cells that were coloured blue are precisely the same cells coloured blue for flow). Data are representative of experiments obtained from a minimum of three separate experiments.

A similar profile of multicellular Ca^2+^ signalling was observed in the endothelium of second‐order mesenteric arteries (Fig. 2 *K* and *L*; see also Supporting information, Movie S2). The magnitude of flow‐induced cellular Ca^2+^ responses in individual endothelial cells was dependent on flow rate (Fig. 3 *A–C*), ceased rapidly upon termination of flow (Fig. 3 *D* and *E*) and increased in a stepwise manner in response to stepwise increases in flow (Fig. 3 *E*). Despite the persistence of complex endothelial Ca^2+^ signalling during ongoing flow, endothelial Ca^2+^ activity was augmented when exogenous ACh was added to the perfusion solution (100 nm) (Fig. 3 *F*; see also Supporting information, Movie S3). Individual cellular Ca^2+^ levels after exogenous ACh treatment were ranked in a similar order to those after flow activation (i.e. those cells that exhibited the greatest increase in [Ca^2+^]_i_ upon activation with flow also exhibited the largest Ca^2+^ levels after addition of exogenous ACh). The converse was also true (i.e. those cells that responded to flow with the smallest increase in [Ca^2+^]_i_ exhibited the smallest Ca^2+^ levels following addition of exogenous ACh) (Fig. 3 *F*).

### Flow and spontaneous Ca^2+^ signalling

**Figure 4:**
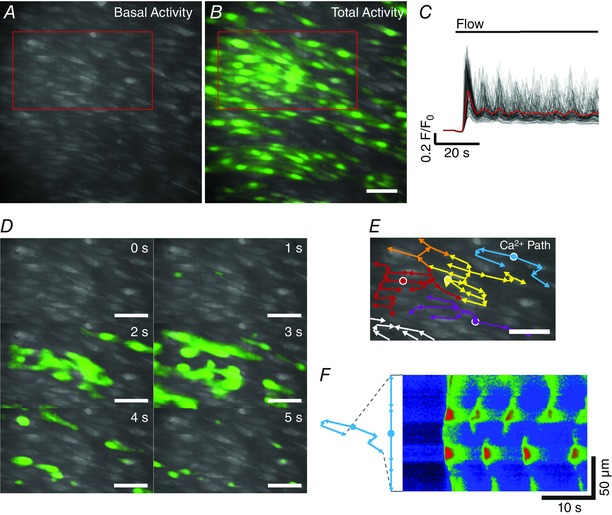
Flow‐evoked whole‐cell Ca^2+^ waves in the endothelium *A*–*B*, representative Ca^2+^ images displaying endothelial cells with basal (*A*) and total flow‐evoked (*B*) activity overlaid in green. *C*, automatically extracted, whole‐cell Ca^2+^ signals from the data shown in (*A*) and (*B*). *D*, time series of images displaying the progression of Ca^2+^ activity as waves from the region of cells outlined in red in (*A*) and (*B*). Large‐scale waves appear to initiate discrete subcellular locations and erupt into large multicellular waves. Data shown in the Supporting information (Movie S4). *E*, flow vectors (coloured arrows) tracing movement of multicellular Ca^2+^ signals from their localized point of origin (white‐outlined circles). In this example, Ca^2+^ signals delineated by the yellow and red arrows merge and continue along a common path (orange). All scale bars = 50 μm. *F*, kymograph image (with distance depicted vertically and time depicted horizontally) formed by measuring Ca^2+^ fluorescence changes (*F*/*F*_0_) along the multipoint line marked in light blue in (*E*) as a function of time following stimulation of the endothelium with fluid flow. As indicated, the multipoint line has been unfolded. The site of initiation of a Ca^2+^ wave is indicated by the dot on the light blue line.

**Figure 5:**
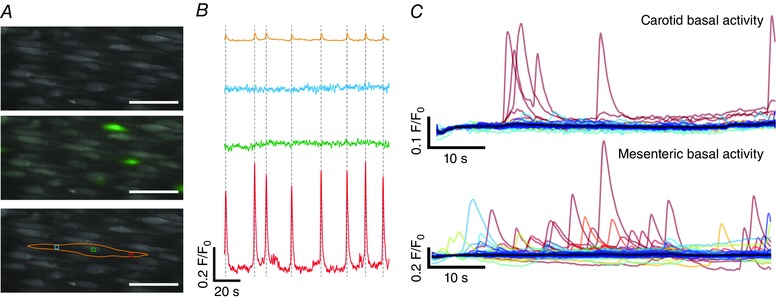
Elementary (local) Ca^2+^ signals in the endothelium *A*, Ca^2+^ image of a region of intact endothelium in an *en face* mesenteric artery (top) with spontaneous activity (in green; middle) and manually‐placed ROIs (bottom), from which Ca^2+^ signals were extracted, overlaid. Spontaneous (i.e. in the absence of stimulation of the endothelium) Ca^2+^ signals occasionally appear in localized, subcellular regions of individual cells. The local signals did not propagate through the cell. *B*, Ca^2+^ signals from the whole endothelial cell (orange) and three subcellular regions (blue, green, red) shown at the bottom of (*A*). The red trace shows repetitive, high intensity Ca^2+^ activity that is not apparent in the signals extracted from other regions of the cell. *C*, basal cellular Ca^2+^ signals extracted from recordings carotid (top) and mesenteric (bottom) artery endothelia using our whole‐cell analysis procedure. Mesenteric artery endothelium showed more spontaneous activity than carotid artery endothelium.

Close inspection of Ca^2+^ imaging recordings revealed that flow‐evoked Ca^2+^ waves originated in discrete subcellular locations, in multiple separate cells across the field‐of‐view (Fig. 4; see also Supporting information, Movie S4). From these locations, Ca^2+^ waves appeared to evolve and spread within and among cells. Studies employing video‐rate imaging have reported spiking, focal increases in Ca^2+^ that occur under basal conditions (Ledoux *et al*. 2008; Sonkusare *et al*., 2012, 2014; Boerman *et al*. 2016). Other studies have reported localized Ca^2+^ waves that may propagate through part or all the cell (Duza & Sarelius, 2004; Kansui *et al*. 2008; Bagher *et al*. 2012). Thus, we investigated whether flow‐evoked Ca^2+^ waves originate in the same locations as spontaneous events. The ability to observe spontaneous Ca^2+^ events in recordings obtained by wide‐field fluorescence microscopy (60 s in duration) was first confirmed by manually placing subcellular ROIs within the boundaries of cells exhibiting spontaneous activity (Fig. 5 *A* and *B*; see also Supporting information, Movie S5). Spontaneous Ca^2+^ events were often apparent in traces obtained from whole‐cell ROIs (Fig. 5 *B*, orange trace) and were observed as restricted waves of Ca^2+^ activity that mostly remained confined within subcellular regions. However, with ∼150 endothelial cells in focus throughout each field‐of‐view, manual and systematic analysis of multiple ROIs for each cell was impractical. Instead, we analysed spontaneous Ca^2+^ events by WAVE Ca^2+^ signal analysis. Using this analysis technique, spontaneously occurring transients were automatically detected in whole‐cell Ca^2+^ traces (Fig. 5 *C*). Spontaneous events were detected infrequently in carotid artery endothelia (1.11 ± 0.54% of cells per artery; *N* = 936 cells, *n* = 7) but frequently in mesenteric artery preparations (20.18 ± 4.68% of cells per artery; *N* = 963 cells, *n* = 6). In those cells displaying spontaneous activity, the frequency of detected events ranged from 0.017 to 0.050 Hz (i.e. one to three events were detected in each of the nine active cells during 60‐second recordings; total of 14 events) in carotid arteries and from 0.017 to 0.083 Hz (i.e. one to five events were detected in each of the 195 active cells during 60 s recordings; total of 304 events) in mesenteric arteries. The mean frequency of detected Ca^2+^ events in active cells was 0.027 ± 0.004 Hz in carotid arteries and 0.026 ± 0.002 Hz in mesenteric arteries.

**Figure 6:**
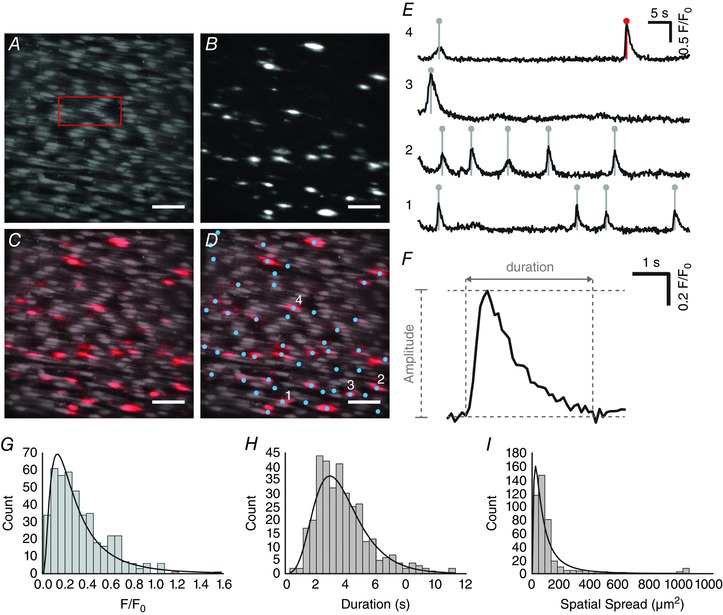
Automated analysis of spontaneous (basal) endothelial Ca^2+^ signalling using FLIKA *A*–*D*, Ca^2+^ images of intact mesenteric artery endothelium: (*A*) averaged fluorescence intensity; (*B*) spontaneous Ca^2+^ activity, visualized by taking the standard deviation of the sequential subtraction image data; (*C*) composite overlay of (*A*) (grey) and (*B*) (red); (*D*) composite Ca^2+^ image with sites of automatically‐detected spontaneous Ca^2+^ activity indicated by blue circles. All image scale bars = 50 μm. *E*, representative Ca^2+^ traces displaying the time course of Ca^2+^ events at the four sites indicated in (*D*). *F*, single spontaneous Ca^2+^ event (indicated in the top trace of *E* by red line/circle) shown on an expanded timescale to illustrate signal metrics. *G*–*I*, histograms of spontaneous Ca^2+^ event data in mesenteric arteries: (*G*) amplitude; (*H*) spatial spread; and (*I*) duration. The duration is calculated as the time taken for the signal to increase from baseline to maximal *F*/*F*_0_ and then from maximal *F*/*F*_0_ back to baseline. Fitted log‐normal distributions (black line) are shown on each histogram.

These results suggest that WAVE Ca^2+^ signal analysis is capable of assessing the observed spontaneous Ca^2+^ signalling. However, recent studies of endothelial Ca^2+^ suggest that whole‐cell measurements may not detect low amplitude subcellular Ca^2+^ events (Socha *et al*. 2012; Dora & Garland, 2013). Furthermore, whole‐cell analyses are incapable of assessing the spatial spread of localized Ca^2+^ events. Thus, we reanalysed spontaneous Ca^2+^ signalling data using a well‐established algorithm (FLIKA) for automated analysis of localized Ca^2+^ events (Fig. 6; see also Supporting information, Movie S5) (Ellefsen *et al*. 2014). Using this alternative analysis method, spontaneous endothelial events were also detected less frequently in carotid arteries (total of 22 event sites; *N* = 936 cells, *n* = 7) than in mesenteric arteries (total of 276 event sites, *N* = 963 cells, *n* = 6). Each of these event sites identified by FLIKA corresponded to a unique endothelial cell. Thus, using this analysis method, on average 2.16 ± 1.38% of endothelial cells displayed spontaneous activity in carotid arteries (*N* = 935 cells, *n* = 7), whereas 28.5 ± 5.3% displayed spontaneous activity in mesenteric arteries, *N* = 963 cells, *n* = 6) .

The frequency of events at each site ranged from 0.017 to 0.1 Hz (i.e. in active cells, one to six events were detected in each of the 22 active cells during 60 s recordings; total 33 events) in carotid arteries and from 0.017 to 0.117 Hz (i.e. one to seven events were detected in each of the 276 active cells during 60 s recordings; total 437 events) in mesenteric arteries. The mean frequency of detected Ca^2+^ events in active cells was 0.012 ± 0.004 Hz in carotid arteries 0.025 ± 0.003 Hz in mesenteric arteries.

**Figure 7:**
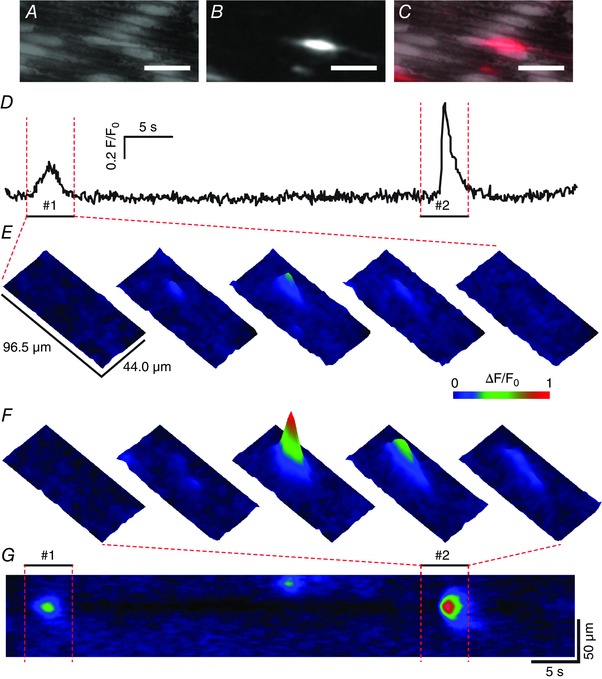
Spatial localization of spontaneous (basal) endothelial Ca^2+^ signalling *A*–*C*, Ca^2+^ images of intact mesenteric artery endothelium corresponding to the red outlined box in Fig. 6 *A*: (*A*) averaged fluorescence intensity showing mesenteric endothelial cells; (*B*) spontaneous Ca^2+^ activity, visualized by taking the standard deviation of the sequential subtraction image data; (*C*) composite overlay of (*A*) (grey) and (*B*) (red). All image scale bars = 25 μm. *D*, Ca^2+^ trace displaying the time course of spontaneous activity at the large site in the centre of *A*–*C*. *E*–*G*, 3‐D surface plots (*E* and *F*) and line scan kymograph (*G*) reveal that some single sites may give rise to Ca^2+^ events that vary substantially in amplitude.

A pooled analysis of all 33 events identified in carotid artery endothelium revealed that the mean event amplitude was 0.17 ± 0.02 *F*/*F*_0_, the mean event duration was 4.05 ± 0.29 s and the mean spatial spread was 145.23 ± 34.87 μm^2^. In mesenteric artery endothelium (437 events), the mean event amplitude was 0.33 ± 0.01 *F*/*F*_0_, the mean event duration was 2.59 ± 0.08 s and the mean spatial spread was 123.07 ± 9.44 μm^2^. Analysis of the distribution of Ca^2+^ events in mesenteric endothelium revealed approximately log‐normal distributions of event amplitudes, durations and spatial spreads (Fig. 6 *G–I*). This analysis shows that the spontaneous Ca^2+^ signals have a non‐quantal, continuous distributions. These results suggest that, under the experimental conditions of the present study, the endothelium displays a continuum of spontaneous subcellular Ca^2+^ waves. By contrast to the propagation of Ca^2+^ waves throughout the whole cell upon activation by flow (Fig. 4), the spatially confined nature of spontaneous waves is evident in Ca^2+^ images (Figs 5 *A*, 6 *A–D* and 7 *A–D*), 3‐D surface plots (Fig. 7 *E* and *F*) and line scan kymographs (Fig. 7 *G*). 3‐D surface plots and kymograph analyses also demonstrate that events arising from a single location may differ in both magnitude and spatial spread (Fig. 7 *E–G*).

**Figure 8:**
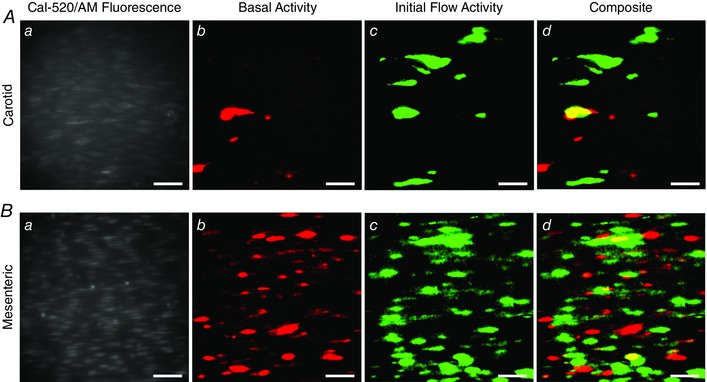
Localization of spontaneous event sites and the sites of origin of flow‐evoked Ca^2+^ waves Ca^2+^ images of carotid (*A*) and mesenteric (*B*) endothelia: (*a*) Ca^2+^ image of endothelium from indicated artery; (*b*) Standard deviation image (STDev; see Methods) showing regions of endothelium that exhibit spontaneous activity; (*c*) STDev image indicating regions of endothelium that first respond to flow; (*d*) overlay of data shown in (*b*) and (*c*). All image scale bars = 50 μm.

To investigate the extent of co‐localization between spontaneous and flow‐evoked Ca^2+^ signals, we compared the spatial distribution of spontaneously occurring events with the spatial distribution of the initial Ca^2+^ response evoked by flow in mesenteric arteries. Some sites of spontaneous activity did give rise to flow‐evoked Ca^2+^ waves (Fig. 8). However, most flow‐evoked Ca^2+^ waves did not appear to originate from sites of spontaneous activity, and not all sites of spontaneous activity appeared to generate flow‐evoked waves (Fig. 8). In mesenteric arteries, 36.0 ± 6.5% (*n* = 5) of the area in which spontaneous endothelial activity occurred overlapped with flow‐evoked Ca^2+^ wave origination sites, whereas 13.95 ± 2.2% (*n* = 5) of the area in which flow‐evoked Ca^2+^ waves originated overlapped with sites of spontaneous activity. These results suggest that some regions of spontaneous activity may give rise to flow activation.

### Pentoject inhibits the endothelial flow response

**Figure 9:**
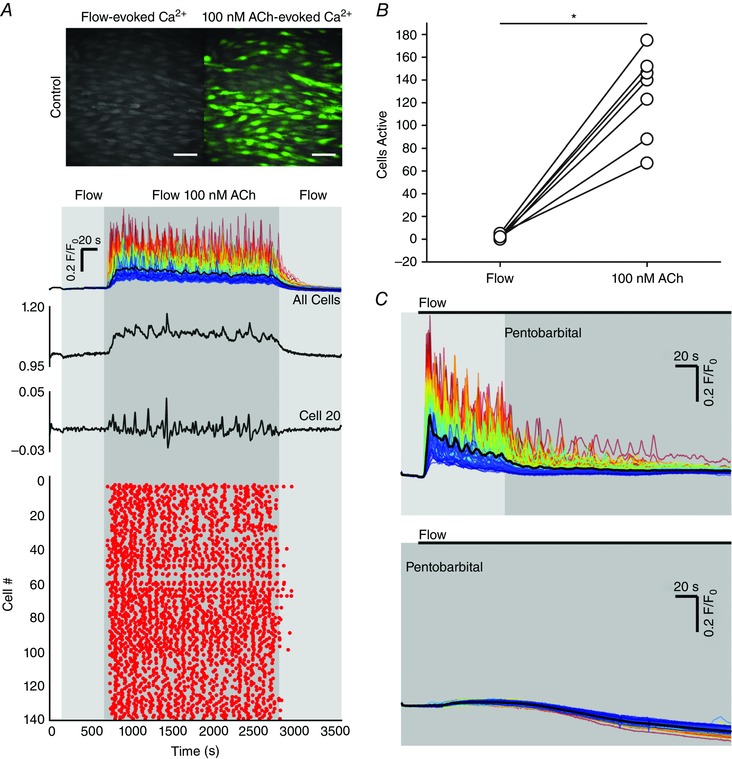
Effects of the barbiturate, pentobarbital, on flow‐ and endogenous ACh‐evoked Ca^2+^ signals *A*, representative endothelial Ca^2+^ images in arteries from animals killed by sodium pentobarbital (Pentoject) do not exhibit a flow‐induced (1.5 ml min^−1^) Ca^2+^ response (left) but the same preparation do respond to ACh (right) with widespread Ca^2+^ signalling. Below the Ca^2+^ images in (*A*), top to bottom: Ca^2+^ responses obtained from all cells across the field‐of‐view both to flow (no response) and ACh; a Ca^2+^ trace from a single cell; the corresponding derivate Ca^2+^ trace; and a rastergram display of Ca^2+^ activity for all cells. Although the entire field of cells responded to ACh with Ca^2+^ changes, there was no response to flow. *B*, paired data from seven separate experiments show the absence of a flow response in animals killed by pentobarbital sodium and normal response to ACh. *C*, in animals killed by CO_2_, the normal Ca^2+^ response to flow was abolished by the addition of the pentobarbital to the perfusion solution. Upper: pentobarbital was added at the point indicated and inhibited the Ca^2+^ increases. Lower: a subsequent period of flow 20 min later evoked no significant flow‐evoked Ca^2+^ increase.

Some previous studies of flow‐mediated dilatation of intact arteries required the presence of ATP in the perfusion solution to obtain consistent responses (Liu *et al*., 2004, 2006). Others studies have not required the presence of extracellular ATP (Falcone *et al*. 1993; Koller *et al*. 1994; Muller *et al*. 1999; Wang *et al*. 2015). We aimed to identify why we observed robust and consistent flow‐mediated endothelial Ca^2+^ responses in the absence of extracellular ATP. Barbiturates have previously been reported to influence endothelial function (Gerkens, 1987; de Wit *et al*. 1999). Thus, we hypothesized that the method of animal dispatch may influence flow‐mediated endothelial responses. Therefore, we studied flow‐mediated endothelial Ca^2+^ signalling in the endothelium of carotid arteries obtained from animals sacrificed by pentobarbital sodium. Importantly, flow‐evoked endothelial Ca^2+^ signalling was absent in arteries obtained from animals killed by the pentobarbital sodium, Pentoject (2.04 ± 0.47% cells responded; *n* = 7) (Fig. 9 *A* and *B*). In these same arteries, the majority of cells responded to exogenous ACh (89 ± 0.13%; 100 nm; *n* = 7) (Fig. 9 *B*). Moreover, flow‐mediated endothelial Ca^2+^ signalling in the endothelium of carotid arteries obtained from rats killed by CO_2_ was abolished by the addition of pentobarbital to the perfusion solution (Fig. 9 *C*). By contrast, another brand of pentobarbital sodium, Euthatal, did not inhibit flow‐evoked endothelial Ca^2+^ signalling when used for animal dispatch (100 ± 0% of cells responded to flow; *n* = 6). These experiments suggest that some formulations of pentobarbital sodium abolish flow‐mediated Ca^2+^ signalling but not the response to exogenous ACh, perhaps because of the different percipients used.

## Mechanisms underlying flow‐evoked Ca^2+^ signalling

### Both Ca^2+^ release and Ca^2+^ entry contribute to flow‐induced endothelial Ca^2+^ signals

**Figure 10:**
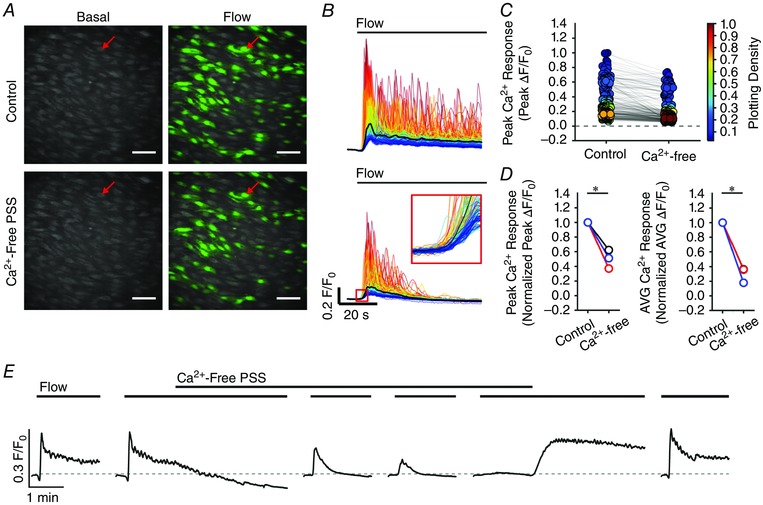
Ca^2+^ release and Ca^2+^ entry contribute to flow‐mediated endothelial Ca^2+^ signalling *A*, representative Ca^2+^ images displaying endothelial activity, before (left) and during (right) flow in the presence (top) and absence (bottom) of extracellular Ca^2+^ in the perfusion solution. Images are from a single artery; the red arrows point to the same individual endothelial cell in each of the images. Scale bars = 50 μm. *B*, flow‐induced Ca^2+^ traces from cells in the images shown in (*A*). Individual traces are coloured according to the magnitude of the peak response under control conditions and the average is overlaid in black. After 20 min in Ca^2+^‐free PSS (*B*, lower trace), all cells still respond to flow with an increase in [Ca^2+^]_i_ (red outlined inset). *C*, plots of the data from the single experiment shown in (*A*) and (*B*), paired responses (peak Δ*F*/*F*_0_ values) measured from the same individual cells in the presence and absence of external Ca^2+^ are shown. Individual data points are coloured according to their plotting density. *D*, paired summary data illustrating changes in peak (left) and time‐averaged (AVG; right) Δ*F*/*F*_0_ values, averaged across individual cells and normalized to control responses per experiment. *E*, representative traces showing the full‐field average Ca^2+^ signals demonstrating the effect of Ca^2+^ removal on flow‐induced Ca^2+^ signals. In Ca^2+^‐free PSS, responses are shown at 10, 20 and 30 min. Flow‐evoked Ca^2+^ signals returned to control levels after ∼10 min when extracellular Ca^2+^ was restored. Note that baseline *F*/*F*_0_ values in (*E*) in Ca^2+^‐free PSS were moved to control levels (dotted line) for clarity and to permit comparison with control responses. ^*^ *P* < 0.01 *vs*. control.

**Figure 11:**
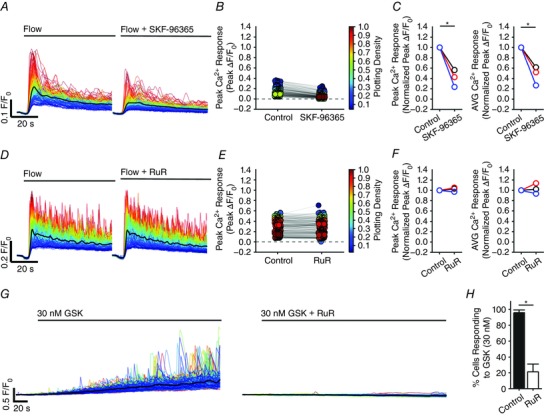
Involvement of TRP channels in flow‐mediated endothelial Ca^2+^ signalling Effects of the TRPC channel inhibitor, SKF‐96365 (50 μm; *A*–*C*), or the TRPV channel inhibitor, RuR (5 μm; *D*–*F*) on flow‐mediated endothelial Ca^2+^ signalling. *A* and *D*, flow‐induced cellular Ca^2+^ traces in the absence (left) and presence (right) of the indicated compound. Ca^2+^ traces are coloured according to the magnitude of the first peak in the control response. *B* and *E*, paired peak Δ*F*/*F*_0_ responses from the data shown in (*A*) and (*D*). *B* and *E*, the peak Ca^2+^ response on precisely the same cells in the absence and presence of the inhibitors. *C* and *F*, paired summary data illustrating peak (left) and time‐average (right) Δ*F*/*F*_0_ values in the absence and presence of the blockers. Responses have been averaged across individual cells and normalized to control responses per experiment. *G*, effect of RuR (5 μm) on Ca^2+^ increases stimulated by the selective TRPV4 channel antagonist, GSK1016790A (100 nm). *H*, summary data illustrating the effect of RuR on the percentage of cells activated by 30 nm GSK1016790A. ^*^ *P* < 0.01 *vs*. control.

In endothelial cells, there are two main mechanisms responsible for an increase in cytosolic Ca^2+^: (1) release of Ca^2+^ from the intracellular store (i.e. the endoplasmic reticulum) and (2) influx of Ca^2+^ across the plasma membrane. To examine the contributions of Ca^2+^ entry and Ca^2+^ release to flow‐induced endothelial Ca^2+^ signalling, we first examined responses in the absence of Ca^2+^ in the perfusion solution (Fig. 10). In these experiments, we first recorded endothelial Ca^2+^ responses to flow of normal PSS (control) and then exchanged the PSS for Ca^2+^‐free PSS that contained EGTA (1 mm; 10 min incubation). Flow‐evoked endothelial Ca^2+^ signalling persisted in Ca^2+^‐free PSS (*n* = 3) (Fig. 10). However, in contrast to the maintained responses obtained in the presence of external Ca^2+^, [Ca^2+^]_i_ returned to baseline levels ∼30 s after the onset of flow (Fig. 10 *B* and *E*). Indeed, as shown by whole field averages, the sustained phase of the flow‐evoked response was lost upon removal of Ca^2+^ from the bath and [Ca^2+^]_i_ levels fell to below baseline levels (Fig. 10 *E*). There was also a gradual reduction in the amplitude and then loss of these initial responses with successive stimulation in Ca^2+^‐free PSS (Fig. 10 *E* and Table 1). The decline in each response with successive stimulation probably occurred as a result of the absence of refilling and depletion of Ca^2+^ stores. Indeed, the decreasing responsiveness was not a result of desensitization to flow because: (1) the reintroduction of extracellular Ca^2+^ resulted in an immediate increase in [Ca^2+^]_i_, indicating the activation of Ca^2+^ entry mechanisms and (2) subsequent flow‐evoked responses in the presence of Ca^2+^ were comparable to control levels (Fig. 10 *E* and Table 1). These results suggest that, after the initial flow‐evoked Ca^2+^ release, there was a sustained influx of Ca^2+^ from the extracellular space. In support, the broad‐spectrum transient receptor potential (TRP) canonical (TRPC) channel antagonist, SKF 96365 (50 μm), abolished the ongoing sustained Ca^2+^entry when applied to the perfusion solution (not shown) but did not abolish subsequent initial flow‐evoked Ca^2+^ signals (Fig. 11 *A–C* and Table 1) (*n* = 3). These results suggest that both Ca^2+^ release from the internal store(s) and store‐operated/receptor‐operated Ca^2+^ ‐entry through TRPC channels contribute to flow‐induced endothelial Ca^2+^ signals, and that Ca^2+^ entry through TRPC channels is required to refill the internal store(s).

TRP vanilloid 4 (TRPV4) channels are reported to contribute to flow‐induced vasodilatation (Hartmannsgruber *et al*. 2007; Mendoza *et al*. 2010; Du *et al*. 2016). However, the role of TRPV4 channels in ACh‐induced vasodilatation is less clear (Köhler *et al*. 2006; Zhang *et al*. 2009; Sukumaran *et al*. 2013; Pankey *et al*. 2014). To test whether TRPV4 channels contributed to the flow‐induced Ca^2+^ signals described, we studied the effects of the the broad‐spectrum TRPV channel antagonist, ruthenium red (RuR) at a concentration (5 μm) five times greater than that required to block both 4 ‐phorbol‐12,13‐didecanoate‐induced TRPV4 currents in intact rat carotid endothelial cells in situ and vasodilatation of pressurized arteries (Köhler *et al*. 2006). RuR did not modify either the initial or sustained component of the flow‐evoked Ca^2+^ responses (Fig. 11 *D–F* and Table 1) (*n* = 3). To confirm that RuR does indeed inhibit endothelial TRPV4 channels, we examined endothelial responses stimulated by the selective TRPV4 agonist, GSK1016790A, in the absence and presence of RuR. It is reported that the endothelium is particularly sensitive and exhibits a particularly steep concentration response to GSK1016790A (in the nanomolar range). Concentrations of 30 nm are reported to stimulate local, subcellular Ca^2+^ events (Bagher *et al*. 2012), whereas higher concentrations (100 nm) induce Ca^2+^ overload in the endothelium (Bagher *et al*. 2012; Sonkusare *et al*. 2012). In these experiments, we used rat carotid arteries from animals that were dispatched by Pentoject and thus exhibited no confounding flow response. GSK1016790A (30 nm) activated 95.8 ± 3.4% of endothelial cells (Fig. 11 *G* and *H*) (*n* = 3). Initially, GSK1016790A (30 nm) caused local Ca^2+^ events (see Supporting information, Movie S6). However, prolonged activation with 30 nm GSK1016790A caused global Ca^2+^ increases and large‐scale propagating Ca^2+^ waves between cells (see Supporting information, Movies S6 and S7), as seen by in other studies using 100 nm GSK1016790A (Bagher *et al*. 2012; Sonkusare *et al*. 2012). Thus, all experiments were terminated after exposure to the TRPV4 agonist. The action of GSK1016790A was inhibited (21.2 ± 10.0% of cells responding; *n* = 3) by pre‐incubation with RuR (5 μm for 20 min). These results suggest that TRPV4 channels contribute little to the flow‐induced endothelial Ca^2+^ signalling described in the present study.

### Flow‐induced Ca^2+^ release occurs via IP_3_ receptors

**Figure 12:**
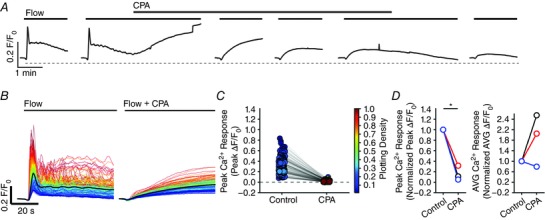
Flow initiates Ca^2+^ release from the endoplasmic reticulum in arterial endothelial cells *A*, representative traces (full field averages) demonstrating the effect of the SERCA inhibitor, CPA (10 μm), on flow‐mediated endothelial Ca^2+^ signalling. *B*, flow‐induced cellular Ca^2+^ traces in the absence (left) and presence (right) of CPA, (*C*) paired peak Δ*F*/*F*_0_ responses (middle) and (*D*) paired summary data illustrating changes in peak (left) and time‐average (right) Δ*F*/*F*_0_ values, averaged across individual cells and normalized to control responses per experiment.^*^ *P* < 0.01 *vs*. control.

To confirm involvement of Ca^2+^ release from internal stores in the flow response, we used the sarcoendoplasmic reticulum ATPase (SERCA) inhibitor, cyclopiazonic acid (CPA) (10 μm), to prevent Ca^2+^ sequestration by the endoplasmic reticulum. The introduction of CPA during sustained flow resulted in an increase in basal [Ca^2+^] (Fig. 12 *A*). Following incubation with CPA (20 min, no flow), basal [Ca^2+^] remained at a steady, elevated level, presumably as a result of activation of store‐operated Ca^2+^‐entry. However, subsequent re‐initiation of flow still triggered an increase in endothelial [Ca^2+^]_i_ (Fig. 12). This increase was slower in onset and more sustained than that obtained in control responses, suggesting that the initial transient phase of flow‐induced Ca^2+^ signals had been abolished. Analysis of the Ca^2+^ responses of individual cells confirmed that the initial transient component present in control responses was lost in the majority of cells (Fig. 12 *B–D* and Table 1) (*n* = 3). All cells did respond with a slow and sustained increase in [Ca^2+^] (Fig. 12 *D*) (*n* = 3), indicative of Ca^2+^ entry. These data, along with the persistence of transient flow‐mediated Ca^2+^ signalling in Ca^2+^‐free PSS, suggest that flow‐induced Ca^2+^ signals result from the interplay of two separate components: a fast transient increase in [Ca^2+^]_i_ as a result of the release of Ca^2+^ from the endoplasmic reticulum, as well as a slower sustained increase in [Ca^2+^]_i_ as a result of Ca^2+^ entry from the extracellular space.

**Figure 13:**
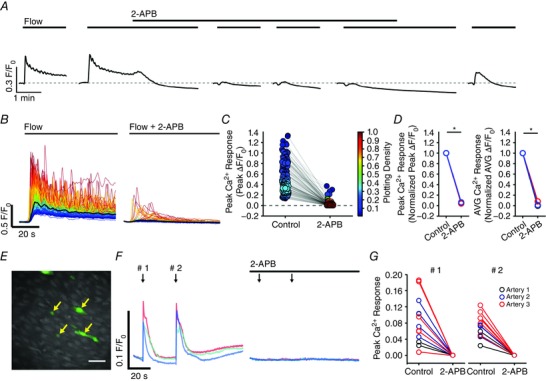
IP_3_ controls flow‐mediated Ca^2+^ release from arterial endothelial cells *A*–*D*, effects of the IP_3_ receptor inhibitor, 2‐APB (100 μm), on flow endothelial whole‐cell Ca^2+^ signals. *A*, representative traces (full field averages) demonstrating the effect of 2‐APB on flow‐evoked Ca^2+^ signals. Paired peak Δ*F*/*F*_0_ responses (*B*) (middle) and paired summary data illustrating changes in peak (*C*) (second from right) and time‐average (right) Δ*F*/*F*_0_ values, averaged across individual cells and normalized to control responses per experiment. Ca^2+^ traces are coloured according to the magnitude of the first peak in the control response. *E*–*G*, effect of 2‐APB on Ca^2+^ signals evoked by local photolysis of caged IP_3_. *E*, Ca^2+^ image with activated cells indicated by yellow arrows. *F*, Ca^2+^ signals, corresponding to the image shown in (*E*) in the absence (left) and presence of 2‐APB (100 μm). *G*, paired summary data illustrating the effect of 2‐APB on IP_3_‐evoked Ca^2+^ signals, technical replications (cells from an individual artery) are grouped and colour‐coded. ^*^ *P* < 0.01 *vs*. control.

**Figure 14:**
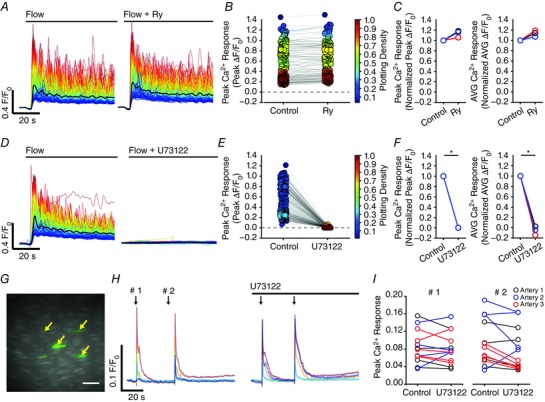
PLC controls flow‐mediated Ca^2+^ release from arterial endothelial cells Effects of Ry (30 μm; *A*–*C*), or the PLC inhibitor, U73122 (5 μm; *D*–*F*) on flow‐mediated endothelial Ca^2+^ signalling. *A* and *D*, flow‐induced cellular Ca^2+^ traces in the absence (left) and presence (right) of the indicated compound. Ca^2+^ traces are coloured according to the magnitude of the first peak in the control response. *B* and *E*, paired peak Δ*F*/*F*_0_ responses from the data shown in (*A*) and (*D*). *B* and *E*, showing the peak Ca^2+^ response on precisely the same cells in the absence and presence of the inhibitors. *C* and *F*, paired summary data showing peak (left) and time‐average (right) Δ*F*/*F*_0_ values in the absence and presence of the blockers. Responses have been averaged across individual cells and normalized to control responses per experiment. *G*–*I*, effect of U73122 on Ca^2+^ signals evoked by local photolysis of caged IP_3_. Two control caged IP_3_ releases (arrows) were followed release of caged IP_3_ in the presence of U73122. *G*, Ca^2+^ image with activated cells indicated by yellow arrows. *H*, Ca^2+^ signals, corresponding to the image shown in (*G*) in the absence (left) and presence of U73122 (100 μm). *I*, paired summary data illustrating the effect of U73122 on IP_3_‐evoked Ca^2+^ signals, technical replications (cells from an individual artery) are grouped and colour‐coded. ^*^ *P* < 0.01 *vs*. control.

In endothelial cells, Ca^2+^ release from the intracellular store(s) may occur via IP_3_ receptors (IP_3_Rs) or ryanodine (Ry) receptors (RyRs) or both (Mumtaz *et al*. 2011). To examine the contribution of IP_3_Rs and RyRs to flow‐evoked Ca^2+^ signalling, we examined the effects of the IP_3_R antagonist, 2‐aminoethoxydiphenyl borate (2‐APB) (100 μm) and the selective RyR modulator, ryanodine (Ry) (30 μm). In addition to being a potent inhibitor of IP_3_‐mediated Ca^2+^ release, 2‐APB is also reported to inhibit Ca^2+^ influx in a number of cell types (Bootman *et al*. 2002). Indeed, exposure to 2‐APB resulted in an immediate fall in [Ca^2+^]_i_ to below baseline levels (Fig. 13 *A*). However, unlike in Ca^2+^‐free PSS (Fig. 10), subsequent initiation of flow failed to induce either transient or sustained increases in [Ca^2+^]_i_ in the majority of cells (Fig. 13 *B–D* and Table 1) (*n* = 3), suggesting that 2‐APB inhibits Ca^2+^ entry channels, as well as Ca^2+^ release via the IP_3_R, in the endothelium of intact arteries. To confirm that 2‐APB inhibits endothelial IP_3_Rs, we examined the effects of 2‐APB on Ca^2+^ signals evoked by flash‐releasing a photo‐activatable form (caged) of IP_3_ in specific endothelial cells. Localized, subcellular, photo‐release of caged IP_3_ generated rapid, repeatable Ca^2+^ transients in the targeted endothelial cells (Fig. 13 *E–G*). However, after 2‐APB (100 μm), photo‐release of caged IP_3_ evoked no Ca^2+^ response (Fig. 13 *E–G*) (*n* = 15 cells, *n* = 3). 2‐APB also did not prevent Ca^2+^ increases stimulated by the selective activator of TRPV4 channels, GSK1016790A (96.2 ± 3.8% of cells responded; *n* = 3). Thus, 2‐APB reliably blocks IP_3_Rs in native endothelium. We did not use xestospongin because, in our experience in smooth muscle cells, xestospongin C does not block Ca^2+^ increases evoked by selective activation of IP_3_Rs using caged IP_3_ (J.G. McCarron, unpublished observations). Other studies have also found that neither xestospongin C, nor xestospongin D blocked any subtype of IP_3_R (Saleem *et al*. 2014). Flow‐evoked responses were not reduced, but rather slightly increased, by Ry (Fig. 14 *A–C* and Table 1) (*n* = 3), suggesting that RyRs contribute little to the observed flow‐evoked responses.

To further examine the contribution of IP_3_, we inhibited phospholipase C (PLC)‐dependent IP_3_ production using the PLC inhibitor, U73122 (5 μm). U73122 completely prevented both flow‐mediated Ca^2+^ release and entry (Fig. 14 *D–F*) in carotid artery endothelia. However, the peak amplitude of Ca^2+^ responses evoked by local photolysis of caged IP_3_ (thus bypassing PLC) in the presence of U73122 was 102 ± 13% of those obtained prior to the introduction of U73122 (Fig. 14 *G–I*) (*n* = 13 cells; *n* = 3). Taken together, the data presented thus far suggest that flow‐mediated endothelial Ca^2+^ signalling arises from IP_3_‐mediated Ca^2+^ release from the endoplasmic reticulum and PLC‐dependent (store‐ or receptor‐operated) Ca^2+^ entry.

### ACh mediates flow‐evoked endothelial Ca^2+^ responses

**Figure 15:**
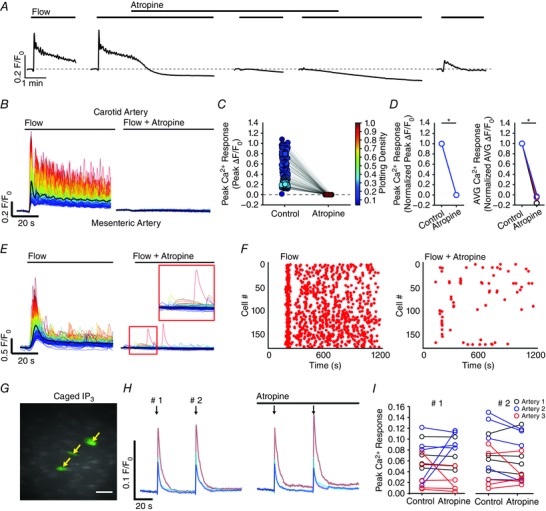
Muscarinic receptor activation contributes to flow‐mediated endothelial Ca^2+^ signalling in large and small artery endothelial cells *A*, representative experiments (full‐field averages) showing the effect of atropine (100 nm) on flow‐evoked (1.5 ml min^−1^) Ca^2+^ signals. *B*–*D*, flow‐induced cellular Ca^2+^ traces (*B*) in the absence (left) and presence (right) of atropine. Paired peak Δ*F*/*F*_0_ responses (*C*) from the data shown in (*B*); the response of each cell paired with itself is shown. Paired summary data (*D*) illustrating changes in peak (left) and time‐average (right) Δ*F*/*F*_0_ values, averaged across individual cells and normalized to control responses per experiment. *E*–*F*, flow‐induced cellular Ca^2+^ traces (*E*) from a mesenteric artery in the absence (left) and presence (right) of atropine (100 nm), and rastergrams of Ca^2+^ activity corresponding to data shown in (*D*). Data in (*E*) to (*F*) are representative of that obtained in three separate experiments. In (*E*), the red outlined inset shows that some small scale Ca^2+^ activity persists in the presence of atropine, despite the lack of a large‐scale response to flow. *G*–*I*, effect of atropine (100 nm) on Ca^2+^ responses stimulated by photolysis of caged IP_3_. *G*, Ca^2+^ image with cells activated by photolysed caged IP_3_ indicated by yellow arrows. *H*, Ca^2+^ signals, corresponding to the image shown in (*G*) in the absence (left) and presence of atropine. *I*, paired summary data illustrating effect of atropine on IP_3_‐evoked Ca^2+^ signals; technical replications (cells from an individual artery) are grouped and indicated by the plot colour. ^*^ *P* < 0.01 *vs*. control.

The inhibition of flow‐evoked endothelial Ca^2+^ signalling by CPA, 2‐APB, and U73122 suggests that the activation of G_q/11 _G‐coupled proteins may form part of an endothelial‐signalling cascade activated by flow. Indeed, the heterogeneity in responses arising from activation of the M_3_ ACh receptor (M_3_AChR) with exogenous ACh was similar of that arising from flow (Fig. 3 *F*). To test whether muscarinic receptor activation contributes to endothelial Ca^2+^ signalling evoked by fluid flow, we examined Ca^2+^ responses in the presence of low concentrations of the muscarinic receptor inhibitor, atropine (100 nm). Atropine caused an immediate fall in endothelial [Ca^2+^]_i_ to below baseline levels (Fig. 15 *A*) and prevented subsequent applications of flow stimulating endothelial Ca^2+^ release or entry in carotid (Fig. 15 *B–D*; see also Supporting information, Movie S8) (*n* = 3). In mesenteric endothelia (Fig. 15 *E* and *F*; see also Supporting information, Movie S9) (*n* = 3), atropine also inhibited large‐scale flow‐mediated Ca^2+^ responses, although the occurrence of more localized flow‐induced Ca^2+^ signals persisted. Additionally, atropine failed to inhibit Ca^2+^ increases as a result of photo‐release of caged IP_3_ (Fig. 15 *G–I*) (peak Δ*F*/*F*_0_, 149± 68% of control; *n* = 14 cells, *n* = 3), suggesting that the block of the flow response in carotid and mesenteric arteries by atropine is a result of the inhibition of muscarinic receptor‐mediated Ca^2+^ release and muscarinic‐receptor mediated store/receptor operated Ca^2+^ entry.

**Figure 16:**
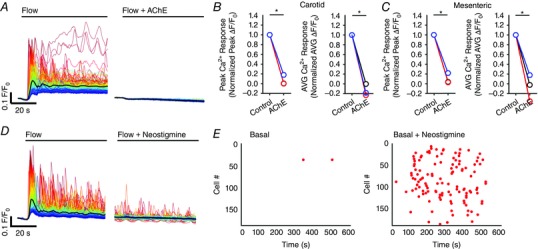
Cholinergic signalling mechanisms govern flow‐mediated endothelial Ca^2+^ signalling in large and small artery endothelial cells *A*, representative experiments demonstrating the effect of AChE (4 U ml^−1^) on flow‐evoked (1.5 ml min^−1^) Ca^2+^ signals. *B*–*C*, paired summary data illustrating changes in peak (left) and time‐average (right) Δ*F*/*F*_0_ values for control and AchE‐treated carotid (*B*) and second‐order mesenteric arteries (*C*) endothelium. Responses have been averaged across individual cells and normalized to control responses per experiment. *D*, flow‐induced cellular Ca^2+^ traces in the absence (left) and presence (right) of neostigmine (10 μm). In (*A*) and (*D*), the black line is the averaged response from all cells in each experiment. *E*, rastergram displays of the activity (peaks in Ca^2+^ activity) of individual cells during under conditions of no flow (basal; left) and no flow in the presence of neostigmine (basal + neostigmine; right). (*D*) to (*E*) are representative of data obtained in three separate experiments. ^*^ *P* < 0.01 *vs*. control.

Because a large variety of G_q/11_ proteins are directly mechanosensitive (Mederos y Schnitzler *et al*. 2008), we next aimed to determine whether muscarinic receptors were activated directly by fluid flow or as a result of the release of endogenous ACh. Therefore, we examined the effects of the hydrolase, AChE (4 U ml^−1^), and the cholinesterase inhibitor, neostigmine (10 μm), on flow‐evoked endothelial Ca^2+^ signalling. AChE abolished flow‐mediated endothelial Ca^2+^ signalling in the endothelium of both carotid (Fig. 16 *A* and *B*) (*n* = 3) and mesenteric (Fig. 16 *C*) (*n* = 3) arteries. Unexpectedly, when neostigmine was present, we no longer observed robust endothelial Ca^2+^ signalling upon commencement of flow (Fig. 16 *D*). However, closer inspection of the data revealed significant basal activity in carotid endothelia (in the absence of flow) after (but not before) neostigmine had been introduced and incubated under no flow conditions for 20 min (Fig. 16 *E*) (*n* = 3). Hence, the endothelium retains endogenous cholinesterase activity. These data, along with the inhibition by AChE, demonstrate that flow‐mediated endothelial Ca^2+^ signalling arises from the release of ACh endogenous to the arterial wall.

**Figure 17:**
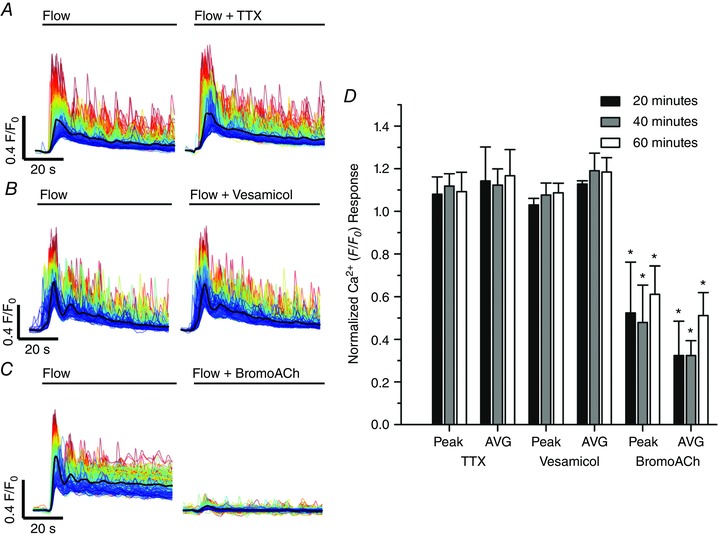
Effect of voltage‐dependent Na^+^ channel block, VAChT inhibition and ChAT inhibition on flow‐mediated endothelial Ca^2+^ signalling in the rat carotid artery *A*–*C*, flow‐induced cellular Ca^2+^ traces in the absence (left) and presence (right) of TTX (*A*) (10 μm), vesamicol (*B*) (10 μm) and bromoACh (*C*) (50 μm) with the averaged response is overlaid in black. *D*, bar graph summarizing normalized peak and time‐averaged (AVG) Ca^2+^ responses after incubation with the indicated compounds for various incubation times (*n* = 3 for each). ^*^ *P* < 0.01 *vs*. control (1; not shown).

We next aimed to investigate whether or not flow‐evoked endothelial Ca^2+^ signalling resulted from the release of neuronal ACh. Thus, we examined endothelial Ca^2+^ responses in the presence of blockers of canonical, neuronal ACh release (Fig. 17). We found that flow‐evoked endothelial Ca^2+^ responses were insensitive to supramaximal concentrations of the voltage sensitive Na^+^ channel inhibitor, TTX (10 μm) (Fig. 17 *A* and *E*) (*n* = 3; IC_50_ = 10 nm) (Zimmer, 2010) and the VAChT inhibitor, vesamicol (10 μm) (Fig. 17 *B* and *E*) (*n* = 3; IC_50_ = 170 nm, Haigh *et al*. 1994). However, flow responses were abolished by the ChAT inhibitor, bromoacetylcholine (bromoACh) (50 μm) (Fig. 17 *C* and *E*) (*n* = 3). Taken together, these results suggest that flow does not activate ACh release from nerves, but rather evokes a non‐vesicular release of ACh from the endothelium.

### Endothelial production of ACh requires pyruvate and mitochondrial production of acetyl‐CoA

ACh is produced when the acetyl group from the coenzyme, acetyl‐CoA, is transferred to choline. Acetyl‐CoA is produced in the mitochondrial matrix, and mitochondrial uncoupling can reduce the supply of cytosolic acetyl‐CoA (Si *et al*. 2009). Therefore, to test whether mitochondrial uncoupling attenuates flow‐evoked endothelial Ca^2+^ signalling, we pharmacologically dissipated the mitochondrial membrane potential (ΔΨ_m_) using the proton uncoupler, carbonyl cyanide 3‐chlorophenylhydrazone (CCCP) (5 μm). The ATP‐synthase blocker, oligomycin (6 μm), was also included to prevent ATP consumption by mitochondria as a result of reversal of ATP‐synthase. In these experiments, we simultaneously monitored endothelial [Ca^2+^]_i_ and ΔΨ_m_ by dual‐loading the endothelium with the Ca^2+^ indicator, Cal‐520/AM, and the mitochondria indicator, TMRE.

**Figure 18:**
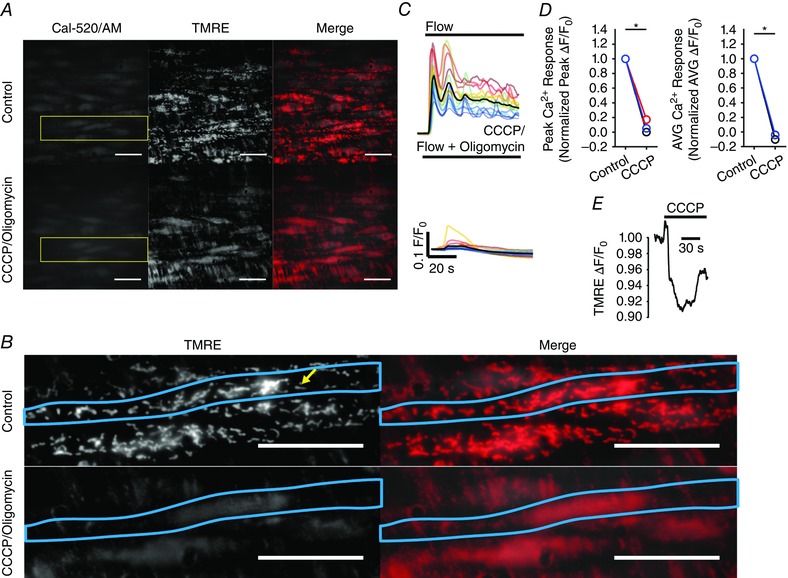
Flow‐induced endothelial Ca^2+^ signalling requires mitochondrial generation of acetyl‐CoA *A*, Ca^2+^ (left), mitochondria (middle) and overlay (right) images of the endothelium of an *en face* artery loaded with Cal 520/AM (5 μm) and TMRE (120 nm) in the absence (top) and presence (bottom) of CCCP (5 μm) and oligomycin (6 μm). Images acquired with a 100× objective. Pharmacological dissipation of the mitochondrial membrane potential with the uncoupler CCCP (used with oligomycin) caused a loss of punctate mitochondrial staining. *B*, scaled images corresponding to the yellow‐outlined box in (*A*), showing punctate mitochondria structure. An individual endothelial cell is demarcated by the blue outline, and an individual mitochondrion is highlighted by the yellow arrow. All image scale bars = 25 μm. *C*, representative flow‐induced cellular Ca^2+^ traces in the absence (top) and presence (right) of CCCP/oligomycin in the perfusion solution. *D*, paired summary data illustrating changes in peak (left) and time‐average (right) Δ*F*/*F_0_* values, averaged across individual cells and normalized to control responses per experiment. *E*, trace of TMRE fluorescence of the individual mitochondria highlighted in (*B*) before and after mitochondrial uncoupling with CCCP (used with oligomycin).

As shown in Fig. 18 *A* and *B*, endothelial mitochondria appear morphologically heterogeneous throughout the cytosol of individual endothelial cells. As in cultured endothelial cells (Shinmura *et al*. 2015) and smooth muscle cells (Chalmers *et al*., 2012, 2015; McCarron *et al*. 2013), multiple morphologies were apparent: small spheres, globules and rods, as well twisting, looped and branched rods. Furthermore, although mitochondria in the nuclear region appeared to be networked, extensive endothelial networks elsewhere in cells were not observed (Fig. 18 *B*). The introduction of a CCCP/oligomycin caused a rapid loss of punctate TMRE staining and an increase in fluorescence throughout the cytoplasm of individual cells (Fig. 18 *A* and *B*), indicative of mitochondrial membrane depolarization and movement of TMRE from mitochondria to the cytoplasm. Significantly, collapsing the mitochondrial membrane potential abolished flow‐evoked endothelial Ca^2+^ signalling (Fig. 18 *C–E*) (*n* = 3). Thus, flow‐evoked ACh production is dependent on polarized endothelial mitochondria.

**Figure 19:**
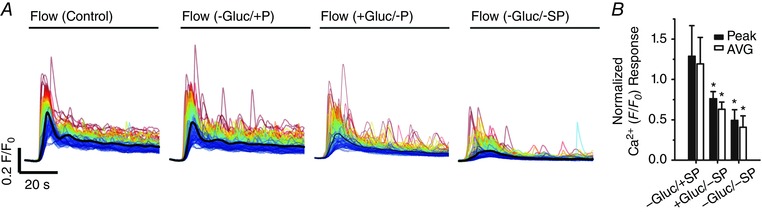
Flow‐induced endothelial Ca^2+^ signalling requires acetyl‐CoA substrates *A*, flow‐induced cellular Ca^2+^ signals obtained from endothelial cells in normal MOPS PSS containing glucose and sodium pyruvate (control), glucose‐free PSS (–Gluc/+SP), pyruvate‐free PSS (+Gluc/–SP) and glucose/pyruvate‐free (–Gluc/–SP) PSS. When glucose was omitted from the PSS, it was replaced with d‐mannitol on an equimolar basis. *B*, bar graph summarizing normalized peak and time‐averaged (AVG) Ca^2+^ responses in response to flow, after incubation in the indicated PSS (*n* = 3 for each). ^*^ *P* < 0.01 *vs*. control (1; not shown).

In mammalian cells, acetyl‐CoA is produced from pyruvate by the pyruvate dehydrogenase complex. Thus far, the experiments described were performed using a standard MOPS PSS, which contained 2 mm pyruvate and 5 mm glucose; the latter may also be used to generate pyruvate during glycolysis. To test whether exogenous pyruvate, or pyruvate derived from glycolysis, contributed to the flow‐evoked endothelial Ca^2+^ responses described, we quantified flow responses in MOPS PSS, and then again after the removal of glucose or pyruvate, or both (Fig. 19). Removal of glucose alone did not significantly affect flow‐evoked endothelial Ca^2+^ signalling. However, removal of pyruvate attenuated flow‐evoked Ca^2+^ responses, and removal of glucose and pyruvate together attenuated responses further still (Fig. 19) (*n* = 3). These results suggest that a sufficient supply of pyruvate is required to enable endothelial production of ACh and that endothelial glycolysis, which may produce sufficient pyruvate, is not necessary for ACh production when exogenous substrates are present.

### ACh release via organic cation transporters mediates the endothelial response to flow

**Figure 20:**
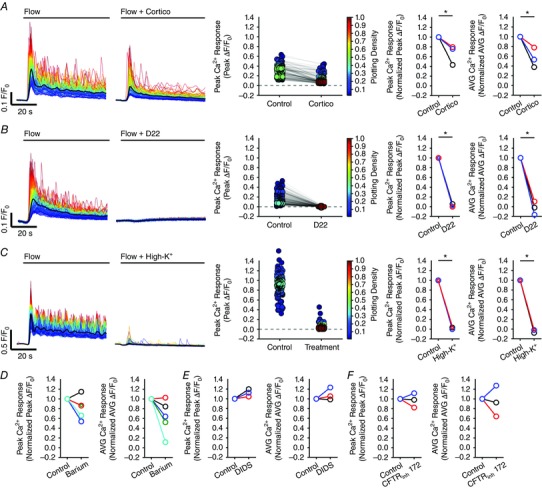
Organic cation transporters mediate electrogenic efflux of ACh *A*–*C*, effects of (*A*) the organic cation transporter inhibitor, corticosterone (cortico; 100 μm); (*B*) the organic cation transporter inhibitor, decynium 22 (D22; 1 μm); and (*C*) high potassium PSS (High‐K^+^) on flow‐mediated endothelial Ca^2+^ signalling. Left: control Ca^2+^ responses from full fields of individual cells, obtained prior to treatment. Ca^2+^ traces are coloured based on the magnitude of the peak control response and the average is overlaid in black. Second from left: Ca^2+^ responses from individual cells, obtained after treatment. The colour of individual cellular Ca^2+^ signals is the same as shown in control responses. Middle: paired responses (peak Δ*F*/*F*_0_ values) from data shown on the left. As a result of contraction to High‐K^+^ PSS, the responses of individual cells are not paired in (*C*). Individual data are points coloured according to their plotting density. Second from right: paired summary data illustrating changes in peak Δ*F*/*F*_0_ values, averaged across individual cells and normalized to control responses per experiment. Right: paired summary data illustrating changes in time‐averaged (AVG; right) Δ*F*/*F*_0_ values, averaged across individual cells and normalized to control responses per experiment. *D*–*F*, summary data illustrating the effects of (*D*) potassium channel blockade with barium (1 mm) and chloride channel blockade with (*E*) DIDS and (*F*) CFTR_inh_172. ^*^ *P* < 0.01 *vs*. control.

**Figure 21:**
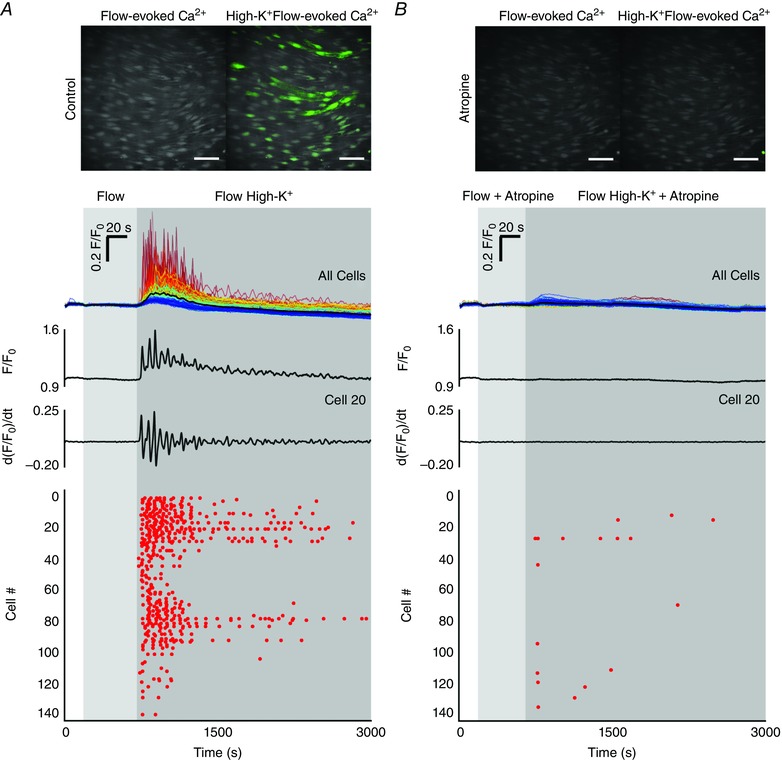
Depolarization‐evoked, atropine‐sensitive endothelial Ca^2+^ signalling *A* and *B*, Ca^2+^ dynamics in endothelial cells that lack flow‐evoked responses (in animals dispatched by Pentojet pentobarbital sodium) exposed to flow and flow of depolarizing (high potassium concentration; High‐K^+^) solutions without (*A*) with (*B*) atropine (100 nm). From top to bottom: fluorescence Ca^2+^ images of carotid artery endothelium during flow (left) and flow of High‐K^+^ PSS (right); the Ca^2+^ responses obtained from all cells across the field‐of‐view; a Ca^2+^ trace and a corresponding derivate Ca^2+^ trace from a single cell; and a rastergram display of Ca^2+^ activity. The grey boxes indicate periods of flow and solution exchange. To prevent smooth muscle cell contraction, nimodipine (10 μm) was present in all solutions. All image scale bars = 50 μm.

Non‐vesicular release of ACh is reported to occur through OCTs (Wessler *et al*. 2001; Bader *et al*. 2014). We found the OCT inhibitors, corticosterone (100 μm; *n* = 3) (Fig. 20 *A*) and decynium 22 (1 μm; *n* = 3) (Fig. 20 *B*) significantly reduced and abolished flow‐mediated endothelial Ca^2+^ signalling, respectively. Efflux of ACh by OCTs is electrogenic (Lips *et al*. 2005). Thus, to examine whether the plasma membrane potential may modulate flow‐mediated endothelial Ca^2+^ responses, we exposed the endothelium to a depolarizing solution (high potassium concentration, 70 mm). Depolarization with high potassium PSS, which does not block endogenous ACh‐mediated endothelial Ca^2+^ signalling (Behringer & Segal, 2015), abolished flow‐mediated endothelial Ca^2+^ signalling (Fig. 20 *C*) (*n* = 3). Depolarizing solutions may also induce Ca^2+^ independent ACh release (Wessler & Steinlein, 1987). Interestingly, in endothelium lacking flow‐evoked responses (in animals dispatched by the pentobarbital sodium, Pentoject), depolarization with high potassium PSS resulted in atropine‐sensitive endothelial Ca^2+^ signalling (Fig. 21). Presumably, high‐K^+^ depolarization resulted in the release of cytosolic ACh stores that could not be released as a result of inhibition of the flow response by Pentoject. These results suggest that Ca^2+^ independent, non‐vesicular endothelial ACh release occurs via OCTs.

An important early endothelial response to flow is the activation of potassium channels that hyperpolarizes the cell (Olesen *et al*. 1988; Cooke *et al*. 1991). This hyperpolarization is reversed to a depolarization by the activation of chloride channels (Barakat *et al*. 1999). Additionally, activation of the Cystic fibrosis transmembrane conductance regulator (CFTR) chloride channel is reported to modulate ACh release in the urothelium (McLatchie *et al*. 2014). We investigated the possibility that potassium channels or chloride channels may initiate the release of endothelial ACh in response to flow. However, we found that flow‐mediated endothelial Ca^2+^ signalling was unaffected by incubation with high concentrations of the potassium channel blocker, barium (1 mm) (Fig. 20 *D*) (*n* = 5), or the chloride channel inhibitors, DIDS (10 μm) (Fig. 20 *E*) (*n* = 3) or CFTR inhibitor 172 (CFTRinh172 (20 μm) (Fig. 20 *F*) (*n* = 3; IC_50_ ∼1 μm) (Kopeikin *et al*. 2010).

### ATP does not mediate flow‐evoked endothelial Ca^2+^ responses

**Figure 22:**
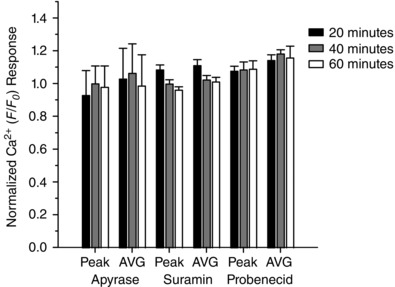
Endogenous ATP does not contribute to flow‐mediated endothelial Ca^2+^ signalling in the rat carotid artery Bar graph summarizing normalized flow‐mediated Ca^2+^ responses after incubation with apyrase (4 U ml^−1^), suramin (100 μm) and probenecid (250 μm) for the incubation times indicated (*n* = 3 each). ^*^ *P* < 0.01 *vs*. control (1; not shown).

The results obtained show that flow activates a muscarinic receptor/PLC/IP_3_R signalling cascade. In the next set of experiments, we aimed to determine whether ATP was involved in the responses observed under the present experimental conditions. To investigate a contribution of ATP, we performed flow experiments with PSS containing the ATPase and ADPase, apyrase, at a concentration sufficient to rapidly degrade any potentially released ATP (4 U ml^−1^) (Shen *et al*. 1992). Additional experiments were performed with the purinergic receptor antagonist, suramin (100 μm; half inhibitor constant on endothelial cells ATP response ∼4 μm) (Guns *et al*. 2005) or the pannexin‐1 hemichannels (a conduit for ATP release in endothelial cells) (Lohman *et al*. 2015) blocker, probenecid (250 μm; IC_50_ 40 μm) (Motais & Cousin, 1976). We found that flow‐evoked Ca^2+^ signals were unaffected by apyrase, suramin or probenecid (Fig. 22) (*n* = 3 each), suggesting that regenerative ATP release does not contribute to the flow‐evoked endothelial Ca^2+^ signals described in the present study.

## Discussion

**Figure 23:**
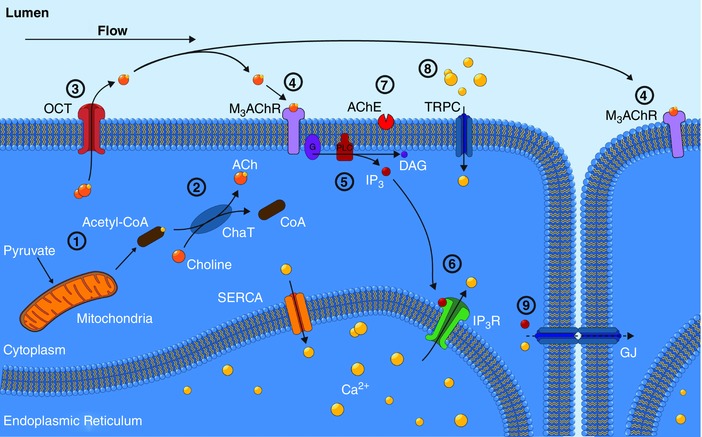
Proposed mechanisms of flow‐mediated endothelial Ca^2+^ signalling (1) Mitochondria generate acetyl‐CoA from pyruvate. (2) ACh is produced, from choline and acetyl‐CoA, in the cytoplasm by ChAT. (3) ACh efflux occurs via OCTs and (4) activates endothelial M_3_AChRs. (5) M_3_AChR activation generates PLC‐dependent IP_3_, which (6) activates IP_3_Rs on the endoplasmic reticulum to release Ca^2+^ from the internal store. Store refilling occurs via the sarcoendoplasmic reticulum (SERCA). (7) The action of endogenous ACh is terminated by membrane bound AChE, but (8) Ca^2+^ responses are maintained during ongoing flow by receptor‐ (DAG) or store‐operated activation of TRPC channels. (9) Gap junction (GJ) hemichannels may also regulate the Ca^2+^ responses by permitting communication via the transmission of Ca^2+^ or IP_3_.

In the present study, using multiple *ex vivo* models, we show that endothelial cells produce and release ACh in response to mechanical activation. An autocrine/paracrine action of endogenous ACh realises a mechanochemical transduction pathway responsible for the physiological phenomena of flow‐mediated endothelial Ca^2+^ signalling in carotid and small mesenteric arteries (Fig. 23) that results in dilatation.

Several lines of evidence support the conclusion that a local endothelial cholinergic signalling mechanism contributes to endothelial mechanochemical coupling. First, similar to the Ca^2+^ response to exogenous ACh (Socha *et al*. 2012; Hill‐Eubanks *et al*. 2014; Wilson *et al*. 2015), fluid flow elicited a biphasic increase in endothelial [Ca^2+^]_i_, which consisted of an initial transient increase in intracellular Ca^2+^ as a result of PLC‐dependent, IP_3_‐mediated release from the endoplasmic reticulum, followed by a sustained elevation of [Ca^2+^]_i_ as a result of Ca^2+^ influx mechanisms. Second, the heterogeneity in endothelial Ca^2+^ signalling initiated by flow was similar to that induced by exogenous ACh. The endothelial response to exogenous ACh is largely dependent upon the muscarinic M_3_AChR (Gericke *et al*. 2011). Finally, flow‐mediated Ca^2+^ responses could be manipulated by interfering with the cholinergic system: low‐concentrations (100 nm) of the muscarinic receptor blocker, atropine, abolished flow‐induced Ca^2+^ activity; AChE, which would degrade any endogenously released ACh, abolished flow‐induced Ca^2+^ activity; and neostigmine, which would prevent the breakdown of locally released ACh, enhanced Ca^2+^ activity. The endothelium expresses VAChT, ChAT and AChE (Parnavelas *et al*. 1985; Arnerić *et al*. 1988; Milner *et al*. 1989; Kirkpatrick *et al*. 2003), and ACh release has been detected from cultured endothelial cells (Kawashima *et al*. 1990; Milner *et al*. 1990; Ikeda *et al*. 1994; Dhein *et al*. 2015) and isolated artery preparations (Zou *et al*. 2015). Importantly, in the latter study, endogenous production of ACh by the endothelium was proposed to contribute to the vasorelaxation induced by hypothermia in the spontaneously hypertensive rat (Zou *et al*. 2015). Fluid flow also induces a nitric oxide‐dependent dilatation of canine coronary arteries that is sensitive to AChE (Martin *et al*. 1996). Together, these observations suggest that ACh mediates flow‐induced mechanosignalling in the endothelium.

## Integration with previous findings

Previous studies suggest that various receptors and ion channels present on endothelial cells may contribute to flow‐mediated vasodilatation (Hill‐Eubanks *et al*. 2014). Genetic ablation of TRPV4 channels in mice results in a diminished flow‐mediated vasodilatation of murine carotid arteries and mesenteric arteries (Mendoza *et al*. 2010). In other studies, flow‐mediated dilatation is impaired in each of kallikrein knockout mice (Bergaya *et al*., 2001, 2004), bradykinin B_2_ receptor knockout mice (Bergaya *et al*. 2001), angiotensin type II receptor knockout mice (Bergaya *et al*. 2004), P_2_X_4_ knockout mice (Yamamoto *et al*. 2006) or P2Y_2_ knockout mice (Wang *et al*. 2015). These studies with various knockout mice suggest that TRPV4 channels, bradykinin B_2_ receptors, angiotensin type II receptors, purinergic P2X_4_ receptors and P2Y_2_ receptors may all be critical mechanosensitive pathways in the endothelium. The contribution of purinergic receptors to endothelial mechanotransduction is supported by evidence that shear stress induces the release of ATP from cultured endothelial cells (Milner *et al*. 1990; Yamamoto *et al*. 2003), freshly isolated endothelial cells (Bodin *et al*. 1991) and pressurized veins (Lohman *et al*. 2015). However, in other studies, robust ATP‐dependent flow responses were observed only in the presence of exogenously added ADP or ATP (Ando *et al*., 1991, 1993; Liu *et al*. 2006). Of direct relevance to the mesenteric artery data reported in the present study, previous investigations of flow‐induced vasodilatation of rat mesenteric arteries have yielded inconsistent results. For example, one study reported that flow‐induced responses were observed in approximately half of the third/fourth‐order mesenteric arteries studied (Liu *et al*. 2004). To increase experimental consistency in flow‐induced responses, in subsequent experiments, extracellular ATP was added to the perfusion solution to ensure that all vessels exhibited flow‐induced responses (Liu *et al*., 2004, 2006). However, further studies from the same research groups reported a complete lack of flow‐induced vasodilatation in mesenteric artery preparations (Winter & Dora, 2007; Beleznai *et al*. 2011). Other investigations in cultured endothelial cells did not observe a significant flow‐induced endothelial Ca^2+^ signals in the absence of ATP (Dull & Davies, 1991; Mo *et al*. 1991). In the present study, flow‐induced Ca^2+^ responses were insensitive to blockade of purinergic receptor or degradation of ATP with apyrase. Similar observations have been made in pressurized cremaster arterioles (Falcone *et al*. 1993), coronary arteries (Muller *et al*. 1999) and rat gracilis muscle arterioles (Koller *et al*. 1994). TRPV4 channels were probably not involved in the flow‐mediated Ca^2+^ signals in the present study because the broad‐spectrum TRPV‐blocker, RuR, at a concentration confirmed to block TRPV4, did not alter the response. Indeed, endogenous ACh‐induced endothelial Ca^2+^ signalling (Wilson *et al*. 2015) and dilatation (Köhler *et al*. 2006) of rat carotid arteries is insensitive to TRPV4 blockade, and normal ACh‐induced dilatation occurs in carotid artery of TRPV4^−/−^ mice (Hartmannsgruber *et al*. 2007).

The diversity of mechanisms reported to explain flow‐induced endothelial responses perhaps reflects different pathways by which shear stress may be transduced by the endothelium and a redundancy required to maintain various short‐ and long‐term vascular responses. Alternatively, the nature of the flow‐mediated Ca^2+^ responses of activated endothelial cells may depend on the magnitude and rate of onset of shear stress (Blackman *et al*. 2000). This latter proposal is significant because blood flow velocity changes in a pulsatile manner as the heart contracts and relaxes and flow rates rise and fall with each heartbeat. It is also tempting to speculate that different mechanisms and signalling pathways may be activated by the various flow regimes associated with constant laminar, pulsatile or turbulent flow. In straight segments of carotid artery (as used in the present study), laminar flow is probably predominant as the major flow pattern. The carotid artery diastolic blood flow rate *in vivo* in anaesthetized rats is ∼2 ml min^−1^ (García‐Villalón *et al*. 1992; Miyashiro *et al*. 1997) and is comparable to the flow rate (1.5 ml min^−1^) used in the present study. It may be that the mechanisms reported in the present study are relevant to the physiological diastolic flow rates.

Flow‐induced endothelial responses have been mostly studied using cultured endothelial cells. In cultured endothelial cells, ACh probably did not contribute to flow‐mediated responses. Exogenous ACh does not stimulate Ca^2+^ signalling in cultured endothelial cells, presumably as a result of a rapid decline in muscarinic receptor expression in culture (Tracey & Peach, 1992). Flow‐mediated Ca^2+^ responses also differ in cultured endothelial cells that are exposed to serum‐containing or serum‐free media (Helmlinger *et al*. 1995), suggesting that serum may also contain Ca^2+^ stimulating endothelial agonists or precursors to endogenous vasoactive molecules.

The question then arises as to what conditions enable flow‐mediated release of endogenous ACh, as well as why the response has not reported been previously. Two results are relevant. First, our data show that flow‐mediated Ca^2+^ signals were inhibited by the pentobarbital sodium, Pentoject. When used for animal dispatch, Pentoject, but not Euthatal, irreversibly abolished the flow response, yet the endothelium still responded to low concentrations of exogenous ACh in these same conditions. Second, the composition of the PSS is critical. Substrates for acetyl‐CoA production are essential for flow‐mediated ACh release to occur. Removal of glucose alone from the PSS did not significantly affect flow‐evoked endothelial Ca^2+^ signalling. However, removal of pyruvate significantly attenuated flow‐evoked Ca^2+^ responses and removal of glucose and pyruvate together almost abolished the response.

Many of the drugs used in the present study have off‐target effects (e.g. 2‐APB) (Bootman *et al*. 2002). For that reason, each conclusion was supported by several interventions. For example, to conclude the internal store was involved in the flow response we used, Ca^2+^‐free PSS, 2‐APB, U73122 and CPA. To conclude that ACh contributes to the response, atropine, AChE and bromoACh and neostigmine were each used. To conclude that ATP does not contribute to the response, we used apyrase, suramin and probenecid. The specificity of many of the drugs used was also confirmed in control experiments with direct activation of the IP_3_R using photolysis of caged IP_3_ or by pharmacological activation of plasma membrane ion channels.

### Spontaneous Ca^2+^ signalling

In the present study, two endothelial Ca^2+^ signalling modalities were apparent after visual inspection of Ca^2+^ video recordings (10 Hz wide‐field recordings; see Supporting information, Movies S1–S9). These were dependent on the artery studied and the mechanisms of activation. Under non‐stimulated (basal) conditions, local Ca^2+^ waves were observed in both carotid and mesenteric endothelia. Spontaneous endothelial activity was observed far less frequently in carotid arteries than in mesenteric arteries. By contrast, activation of the endothelium with fluid flow resulted in whole‐cell propagating Ca^2+^ waves that began in small subcellular regions. Given the sensitivity of the flow response to inhibitors, ACh and IP_3_R are probably the major contributors to the initiation of the flow‐evoked wave.

Spontaneous events of various sizes have previously been reported in the endothelium of pressurized rat carotid (5 Hz imaging: Wilson *et al*. 2016) and third‐order mesenteric arteries (3 Hz imaging: Kansui *et al*. 2008; Bagher *et al*. 2012), as well as in the endothelium of ureteric microvascular networks in situ (20‐50 Hz imaging: Burdyga *et al*. 2003; Borisova *et al*. 2009; Borysova *et al*. 2013). Other studies of rat superior (0.33 Hz imaging: Oishi *et al*. 2001) and second‐order (1 Hz imaging: Lamboley *et al*. 2005) mesenteric arteries did not report the occurrence of spontaneous endothelial Ca^2+^ signalling, whereas it was not observed third‐order mesenteric arteries *ex vivo* (1 Hz imaging: McSherry *et al*. 2005) or mesenteric networks *in situ* (20–30 Hz imaging: Borisova *et al*. 2009). Similarly, significant spontaneous activity was not observed in rat thoracic aorta (0.25 Hz imaging: Jen *et al*., 2000, 2002 *a*,*b*; Huang *et al*. 2001). Spontaneous endothelial Ca^2+^ activity has also been reported in murine mesenteric arteries in *en face* (8 Hz imaging: Francis *et al*. 2012; Qian *et al*. 2014), *en face*/pressurized (15–30 Hz imaging: Ledoux *et al*. 2008) and *in situ* (30 Hz imaging: Boerman *et al*. 2016) preparations, as well as in murine cremaster muscle arterioles *in situ* (5 Hz imaging: Duza & Sarelius, 2004) and in *en face* porcine coronary arteries (Francis *et al*. 2016), although it was not observed in murine aorta (0.33–50 Hz imaging: Marie & Beny, 2002; Boittin *et al*. 2013; Prendergast *et al*. 2014 *a*,*b*). However, it is unclear whether the lack of a description of spontaneous events in some of these studies is a result of their absence, an inability to resolve such events because of insufficient temporal resolution, or because investigative focus was on stimulated Ca^2+^ signalling.

### Characteristics of spontaneous local events

Local IP_3_‐mediated Ca^2+^ events were initially classified into a three‐tier signalling hierarchy (Lipp & Niggli, 1996; Parker *et al*. 1996). The fundamental unit of this hierarchy are fast (<100 ms duration), highly localized (a few um spatial spread), low amplitude (tens of nM) Ca^2+^ release events (‘Ca^2+^ blips’) that result from the opening of a single IP_3_R. The second level includes slightly larger (> 50 nm) and longer lasting (<360 ms) Ca^2+^ release events (‘Ca^2+^ puffs’) that arise from the concerted opening of several IP_3_Rs within a cluster. Ultimately, these elementary Ca^2+^ signals events may co‐ordinate to generate global Ca^2+^ release events, Ca^2+^ waves, throughout the cell. In cultured endothelial cells, the smallest events, Ca^2+^ blips or small Ca^2+^ puffs, are reported to have a mean amplitude of 23 nm and a spread of 1–3 μm and these may precede the occurrence of Ca^2+^ waves (Hüser & Blatter, 1997).

In intact endothelium, two additional elementary Ca^2+^ signals may contribute to endothelial function. Endothelial ‘Ca^2+^ pulsars’ are IP_3_R mediated Ca^2+^ events that occur in murine mesenteric arteries (Ledoux *et al*. 2008). Pulsars are brief (<300 ms) spike‐like Ca^2+^ events, limited in spread to ∼16 μm^2^, that occur at a rate of ∼0.1 Hz preferentially at or near myoendothelial gap junctions (Ledoux *et al*. 2008). TRPV4‐mediated Ca^2+^ sparklets are another type that arise from Ca^2+^ influx rather than release (Sonkusare *et al*. 2012). TRPV4 sparklets were uncovered after store depletion and have defined characteristics (amplitude 0.2 *F*/*F*_0_; spread 11 μm^2^), although they occur infrequently under control (store intact) conditions, with one event occurring only every ∼10 min (∼0.0016 Hz).

In the present study, spontaneous Ca^2+^ events had a very wide spread of amplitudes and durations, and occurred with a range of frequencies (0.05–0.017 Hz). These events ranged from subcellular, local Ca^2+^ waves to whole‐cell Ca^2+^ waves. Very brief Ca^2+^ events (i.e. puffs and pulsars) were not observed, presumably as a result of insufficient temporal resolution. Other studies (Burdyga *et al*. 2003) conducted in endothelial cells have also reported a variety of spontaneous IP_3_R Ca^2+^ waves in intact endothelium that ranged from highly localized subcellular events (i.e. puffs) to Ca^2+^ waves that propagated though part (i.e. abortive waves) (Bootman *et al*. 1997 *b*) or the entirety of the cell (i.e. whole‐cell waves). Each of these events (puffs, abortive waves, waves) may vary in amplitude, frequency and spatial spread. Indeed, as reported in the present study and elsewhere (Burdyga *et al*. 2003), multiple events arising from a single site within a single cell may vary in amplitude and spatial spread. Such a continuum of IP_3_R mediated events have also been described in several studies in other tissues; even individual puffs may extend over a range 20–600 nm in amplitude and 100–600 ms in duration (Bootman *et al*. 1997 *a*; Sun *et al*. 1998; Thomas *et al*. 1998).

In the present study, the continuum of spontaneous endothelial Ca^2+^ waves (amplitude, duration and spread) may be a consequence of the Ca^2+^ imaging method. For the smallest of local events (i.e. blips and puffs; not observed in the present study), the upstroke and amplitude is directly related to both the open time of the release channel and the number of channels that are gated together. By contrast to electrophysiological single channel recordings, a single channel opening for a duration twice as long as a previous channel opening will generate a local Ca^2+^ signal with an amplitude double that of the previous opening (assuming linear buffering/removal). Thus, given the exponential distribution of open times of single channels (Hille, 1992), the amplitude of local Ca^2+^ release amplitudes would have an exponential distribution. For intermediate events (abortive waves) and larger events (whole‐cell waves) as observed in the present study, the amplitude and spread of the signal also depend on the number of channels/clusters recruited. In our experiments, imaged at 10 Hz, we did not limit the progression of a Ca^2+^ signal from one IP_3_R cluster to the next and spontaneous Ca^2+^ events were observed as either abortive or whole‐cell waves. Thus, the distribution of durations, amplitudes, and spread of these spontaneous Ca^2+^ waves presumably arises from variations in: (1) the duration of channel opening times; (2) the number of channels gated; and (3) the extent of recruitment of neighbouring IP_3_R clusters. In addition to these biophysical variables, the methods used to measure cytoplasmic Ca^2+^ (Ca^2+^ indicators, microscope systems, acquisition speeds; analysis methods), the vascular bed under study and the experimental conditions (e.g. method of animal dispatch, PSS composition) probably all determine whether spontaneous events occur and whether they may be resolved.

## Experimental considerations

### Wide‐field microscopy

The experiments described in the present study relied upon wide‐field fluorescence microscopy based imaging to assess endothelial [Ca^2+^]_i_. Using this technique, we were able to image fields of endothelium containing ∼150 cells at 10 Hz and resolve a range of spontaneously occurring subcellular Ca^2+^ waves, and stimulated (flow) whole‐cell propagating waves. This approach is consistent with other endothelial Ca^2+^ imaging studies utilizing confocal microscopy (McSherry *et al*. 2005; Kansui *et al*. 2008; Socha *et al*. 2012). One advantage to using wide‐field imaging is the enhanced depth‐of‐field achieved compared to confocal imaging. In the present study, this permitted focus to be maintained across areas of endothelium encompassing ∼150 cells. Furthermore, a single area of endothelium could be repeatedly imaged for extended periods of time. This enabled us to record a single field of endothelium before, after and even during the introduction and washout of pharmacological compounds. This approach yields vast amounts of information, although the quantity of data generated renders visual identification and manual analysis a laborious and time‐consuming process and places emphasis on the need for automated data analysis techniques.

### Ca^2+^ signal analysis

In the present study, automated data analysis was used because of the large number of cells imaged. Many studies rely on manual visual inspection methods to first identify the presence of Ca^2+^ signals and then position regions of interest for data extraction. This manual approach is laborious but feasible when applied to small numbers of cells (e.g. ∼10) are imaged for relatively short periods of time (e.g. 30 s). However, it is impractical when large numbers of cells (>100) are imaged for long times (e.g. 2 min) where the number of events occurring may run into thousands. The method used in the present study (WAVE Ca^2+^ signal analysis) is a refinement of a previously developed largely‐automated analysis for large‐scale endothelial Ca^2+^ imaging data (Wilson *et al*., 2015, 2016). WAVE Ca^2+^ signal analysis enabled the rapid quantification of flow‐evoked whole‐cell Ca^2+^ signalling metrics from all cells within each field‐of‐view and, importantly, the pairing of individual cell responses across multiple, repeated observations. This analysis method automatically detects cell outlines and assign regions of interest to extract Ca^2+^ changes from all cells in the field (∼150 cells). The method is objective, removes user bias and permits large quantities of data to be processed in a few minutes. However, because it assigns whole‐cell regions of interest (rather than subcellular regions), the analysis may not resolve particularly low amplitude, subcellular Ca^2+^ events. The limitation is not in the visualization of a low‐amplitude event but, instead, is a result of the signal being averaged out because of a lack of a signal elsewhere within the cell (Socha *et al*. 2012). This restriction was not a limiting factor *per se* in the present study because our analysis of the regulation of flow‐evoked Ca^2+^ signalling was focused on the assessment of pharmacological intervention on the resultant whole‐cell Ca^2+^ waves. However, it may have resulted in an under‐reporting of low‐amplitude, spontaneous signals and may have missed some subtleties in the flow‐evoked signalling modality because, for example, whole‐cell Ca^2+^ waves and subcellular events may occur sequentially in the same cell (Socha *et al*. 2012).

To circumvent these limitations, we reanalysed the spontaneous imaging data using a well‐established, pixel‐based automated algorithm for site‐specific analysis of local Ca^2+^ events (Ellefsen *et al*. 2014). Analysis by either means (WAVE or FLIKA) revealed that the proportion of cells exhibiting spontaneous signalling was greater in mesenteric than in carotid artery endothelium. WAVE analysis was simpler and quicker than the pixel‐based analysis. However, FLIKA was more effective in discriminating local events and detected a large number of spontaneous events (∼100) missed by our whole‐cell ROI analysis. Consequently, the frequency of spontaneous events was ∼0.025 Hz in rat mesenteric arteries, in agreement with the values reported using manual analyses of spontaneous activity (Kansui *et al*. 2008; Bagher *et al*. 2012).

### Emergence of Ca^2+^ waves from elementary signals

Endothelial cells form a network of interconnected cells. The network structure and existence of intercellular gap junctions may facilitate the transmission of activity between cells by permitting the passage of IP_3_ and/or Ca^2+^. Such a mode of communication would result in intercellular propagating Ca^2+^ waves. Indeed, whole‐cell latency analysis demonstrates the co‐ordination of endothelial Ca^2+^ signalling in intact endothelial preparations (Socha *et al*. 2012). However, a thorough examination of Ca^2+^ wave activity requires the development of more advanced analyses. Although the present study did not seek to dissect such information, neither whole‐cell ROI, nor pixel‐based analysis of spontaneous events can properly assess the propagation of inter‐ or intracellular Ca^2+^ waves. Indeed, despite being able to distinguish between Ca^2+^ signals arising at a particular site and those resulting from ‘bleed‐through’ from adjacent sites (Ellefsen *et al*. 2014), in a preliminary analysis of flow‐evoked whole‐cell Ca^2+^ waves, the pixel‐based analysis failed to adequately characterize whole‐cell Ca^2+^ responses. Perhaps optimization of pixel‐based analysis for datasets displaying lower intensity, less complex signalling and obtained at a higher rate (e.g. video‐rate) than in the present study (10 Hz), combined with advanced cross‐correlation techniques (Malmersjö *et al*. 2013; Smedler *et al*. 2014), will permit the evolution of endothelial Ca^2+^ signalling, from elementary events (e.g. pulsars) to multicellular Ca^2+^ waves, to be determined.

## Physiological significance

Taken together, the results reported in the present study highlight a novel role for ACh in the regulation of vascular function and establish ACh as an element linking mechanical forces and endothelial control of the blood vessel wall. The present study does not specifically address the *in vivo* physiological consequences of shear stress induced release of ACh from the endothelium. However, it is tempting to speculate on the physiological consequences of flow‐induced ACh release. In all vascular beds, increasing tissue activity results in increased regional blood flow. For example, during exercise, blood flow to skeletal muscle increases in proportion to the metabolic demand of the tissue (Murrant & Sarelius, 2015).  There is an initial increase in blood flow that declines to an elevated steady‐state level. Different mechanisms may generate the initial and sustained responses. In the context of the present study, one intriguing finding shows that exercise‐ or mental stress‐induced increased blood flow in skeletal muscle was abolished by block of muscarinic receptors under some conditions (Matsukawa *et al*. 2013). Several proposals have been made to explain the route by which cholinergic activation of the endothelium may have occurred. For example, ACh ‘spillover’ from motor end plates of the neuromuscular junctions may activate endothelial muscarinic receptors to cause a dilatation that is required for the ascending vasodilatation integral to exercise‐induced hyperaemia (Welsh & Segal, 1997). In support of cholinergic involvement in exercise‐induced hyperaemia, increases in forearm blood flow with exercise are reduced by atropine (Dietz *et al*. 1997). Cholinergic vasodilatation may also contribute to changes in vascular resistance at the onset of isometric handgrip exercise because cholinergic blockade (glycopyrrolate) reduced the fall in vascular resistance (Vianna *et al*. 2015). Intravenous atropine also attenuates the increase in blood pressure, brachial blood flow and brachial vascular conductance in the exercising forelimb during voluntary isometric exercise in the conscious cat (Komine *et al*. 2008). However, the proposed contribution of the cholinergic system in the increased blood flow remains controversial and has been challenged in several studies in humans (Brock *et al*. 1998; Dyke *et al*. 1998) and experimental animals (Donald *et al*. 1970; Buckwalter & Clifford, 1999; Naik *et al*. 1999). It may be that such conflicting findings arise because of differences in exercise modality. Indeed, a local cholinergic component is evident at the onset of isometric handgrip but not dynamic (cycling) exercise (Vianna *et al*. 2015). Interestingly, blood pyruvate measurably increases during and/or after moderate exercise (Yanof, 1943; Ahlborg & Felig, 1982; Lundgren *et al*. 1988; Henderson *et al*. 2004). On the basis of the present findings, increases in the ACh precursor, pyruvate, may be expected to increase endothelial generation of ACh and thus contribute to reactive hyperaemia.

## Summary and perspective

In addition to regulating artery diameter, flow‐mediated shear stress on the endothelium regulates several other vascular responses. Shear stress may act on the endothelium to control angiogenesis, vascular remodelling (Lucitti *et al*. 2007) and the occurrence of disease such as atherosclerosis (Gibson *et al*. 1993). Several studies suggest that cholinergic signalling mechanisms and ACh release may contribute to these responses. For example, AChE inhibition accelerates endothelial cell migration (Cooke, 2007) and angiogenesis branch formation (Dhein *et al*. 2015). These results suggest that local ACh release may modulate endothelial cell migratory and proliferative capacity. Endothelial ACh release may therefore have significance in vascular control beyond the regulation of contractile function.

## Additional information

### Competing interests

The authors declare that they have no competing interests.

### Author contributions

CW and JMG designed the experiments. CW and ML performed the experiments. CW analysed the results. The manuscript was drafted by CW and JGM. The manuscript was prepared and written with contributions from all authors. All authors have approved the final version of the manuscript and agree to be accountable for all aspects of the work. All persons designated as authors qualify for authorship, and all those who qualify for authorship are listed.

### Funding

This work was funded by the Wellcome Trust (092292/Z/10/Z and 202924/Z/16/Z) and the British Heart Foundation (PG/11/70/29086), whose support is gratefully acknowledged.

## Supporting information

Disclaimer: Supporting information has been peer‐reviewed but not copyedited.

**Movie S1**. Flow‐mediated Ca^2+^ signals in the endothelium of a carotid artery. Ca^2+^ signalling was examined in a cut‐opened (*en face*) preparation of an artery in which the endothelium was loaded with the Ca^2+^ indicator, Cal‐520/AM. The movie shows a raw 5 min long Ca^2+^ imaging recording (left; Played at 10× speed) and baseline‐corrected Ca^2+^ signals (*F*/*F*_0_) for a single cell (light blue, cell indicated by arrow head) and for the whole field‐of‐view (red). The timescale of the Ca^2+^ imaging recording is indicated at the top right of the movie panel. Flow (1.5 ml min^−1^) was initiated at approximately *t* = 15 s. Scale bar = 50 μm.

**Movie S2**. Flow‐mediated Ca^2+^ signals in the endothelium of a second‐order mesenteric artery. Ca^2+^ signalling was examined in a cut‐opened (*en face*) preparation of an artery in which the endothelium was loaded with the Ca^2+^ indicator, Cal‐520/AM. The timescale of the Ca^2+^ imaging recording is indicated at the top right of the movie panel. Flow (1.5 ml min^−1^) was initiated at approximately *t* = 15 s. Scale bar = 50 μm.

**Movie S3**. Exogenous ACh augments flow‐mediated Ca^2+^ signalling. Ca^2+^ signalling was examined in a cut‐opened (*en face*) preparation of an carotid artery in which the endothelium was loaded with the Ca^2+^ indicator, Cal‐520/AM. The timescale of the Ca^2+^ imaging recording is indicated at the top right of the movie panel. Flow (1.5 ml min^−1^) was initiated at approximately *t* = 15 s and ACh (100 nm) was added at approximately *t* = 15 s. The movie corresponds to data shown in Fig. 3 *F*. Scale bar = 50 μm.

**Movie S4**. Flow‐mediated Ca^2+^ signals originate in distinct subcellular locations. The movie shows active Ca^2+^ wavefronts (green; obtained by sequential subtraction of raw imaging data) overlaid on a single Ca^2+^ image (grey; obtained by averaging raw imaging data). The movie begins at the onset of flow, and is first played before being rewound and played at double speed. The movie corresponds to data shown in Fig. 4. Scale bar = 50 μm.

**Movies S5**. Spontaneous Ca^2+^ signals in the endothelium of a mesenteric artery. Ca^2+^ signalling was examined in a cut‐opened (*en face*) preparation of an artery in which the endothelium was loaded with the Ca^2+^ indicator, Cal‐520/AM. The movie shows a raw 60 s long Ca^2+^ imaging recording (left) and a binary image highlighting locations of spontaneous activity. In the binary image, pixels corresponding to a single event arising from a single location are all displayed with the same colour. Scale bar = 50 μm.

**Movie S6**. Activation of TRPV4 channels induces local Ca^2+^ signals followed by global Ca^2+^ increases in *en face* carotid artery preparations. The movie begins just prior to the onset of activation (*t* = 20 s) by the specific TRPV4 agonist, GSK1016790A (30 nm). Scale bar = 50 μm.

**Movie S7**. Prolonged activation of TRPV4 channels induces large‐scale propagating Ca^2+^ waves in *en face* carotid artery preparations. The movie shows active Ca^2+^ wavefronts (green; obtained by sequential subtraction of raw imaging data) overlaid on a single Ca^2+^ image (grey; obtained by averaging raw imaging data). Scale bar = 50 μm.

**Movie S8**. Flow‐mediated Ca^2+^ signalling in carotid artery endothelia is abolished by atropine. The movie shows three sequential experimental recordings obtained from a single *en face* carotid artery preparation. The first recording shows a control response to flow (flow was initiated at *t* = 15 s). The second recording shows a control response during which atropine (100 nm) was added to the perfusion solution (flow was initiated at *t* = 20 s and atropine was introduced at *t* = 115 s). The third recording shows a response to flow in the presence of atropine (after a 20 min incubation; flow was initiated at *t* = 20 s). Scale bar = 50 μm.

**Movie S9**. Flow‐mediated Ca^2+^ signalling in mesenteric artery endothelia is abolished by atropine. The movie shows two sequential experimental recordings obtained from a single *en face* carotid artery preparation. The first recording shows a control response to flow (flow was initiated at *t* = 17 s). The second recording shows a response to flow in the presence of atropine (100 nm; after a 20 min incubation). Large‐scale, flow‐mediated Ca^2+^ signalling is absent following atropine treatment. Scale bar = 50 μm.
